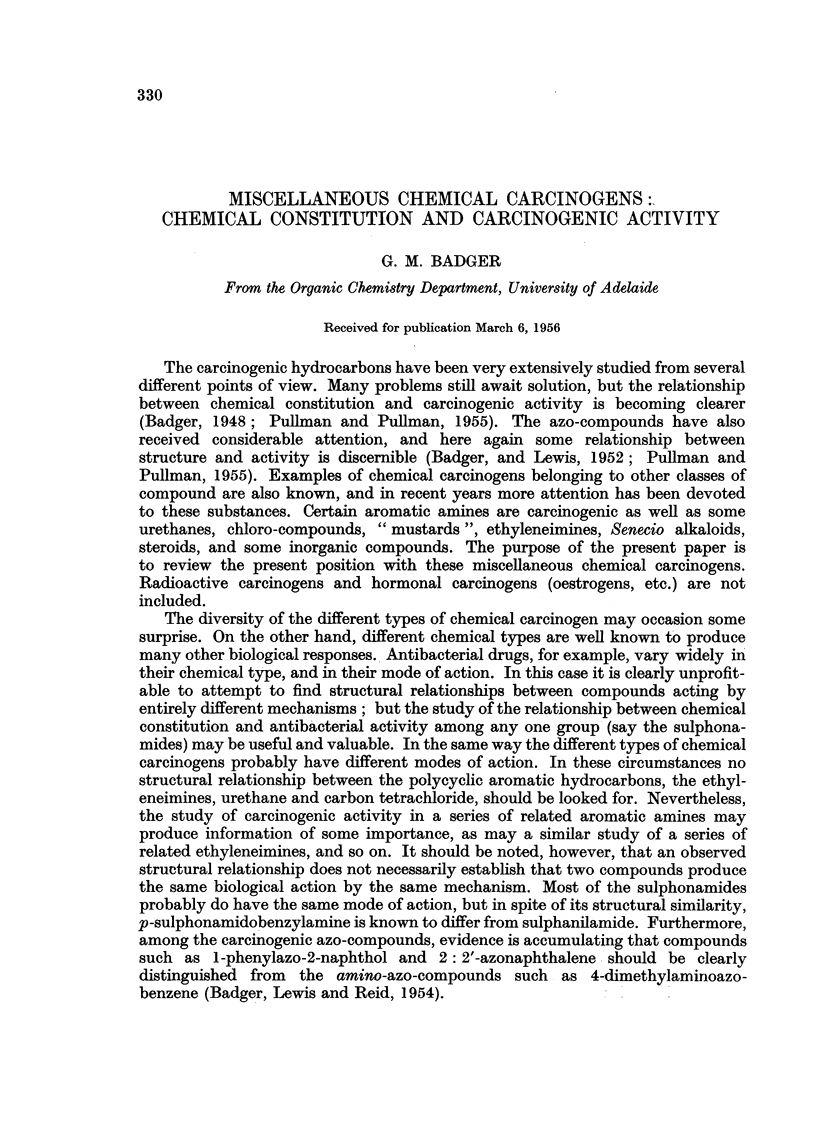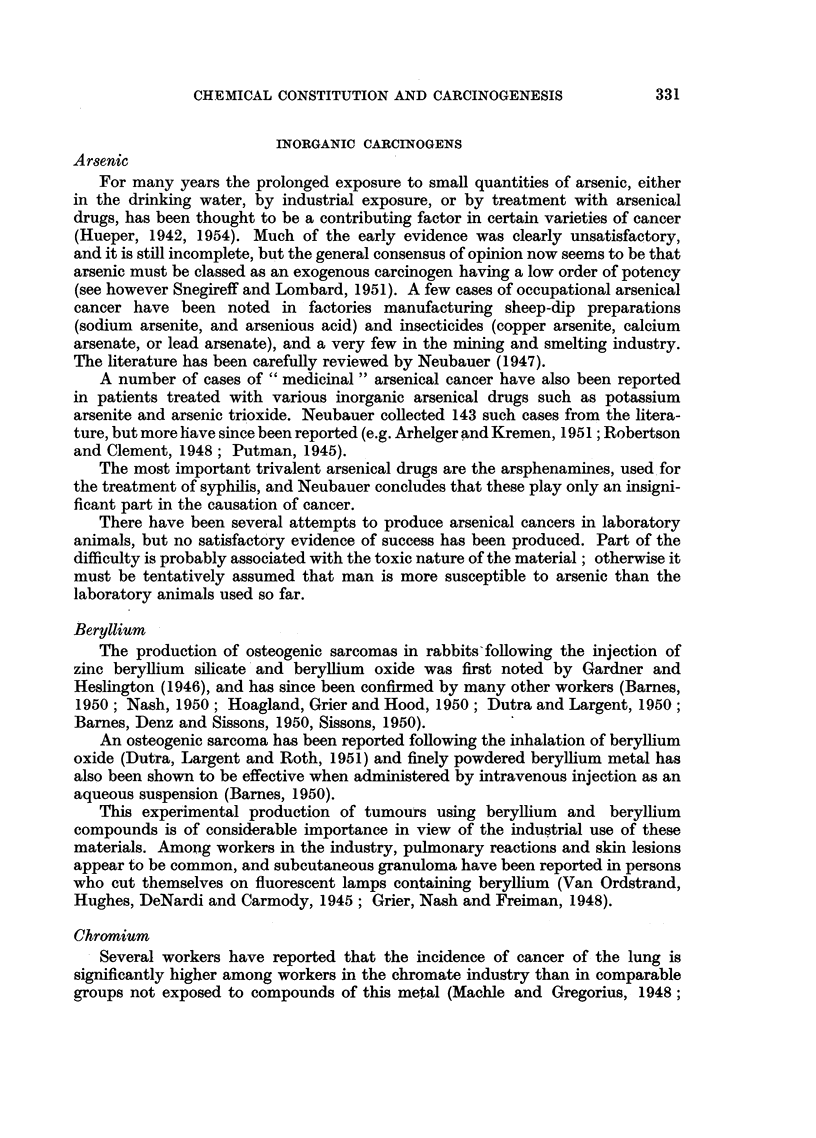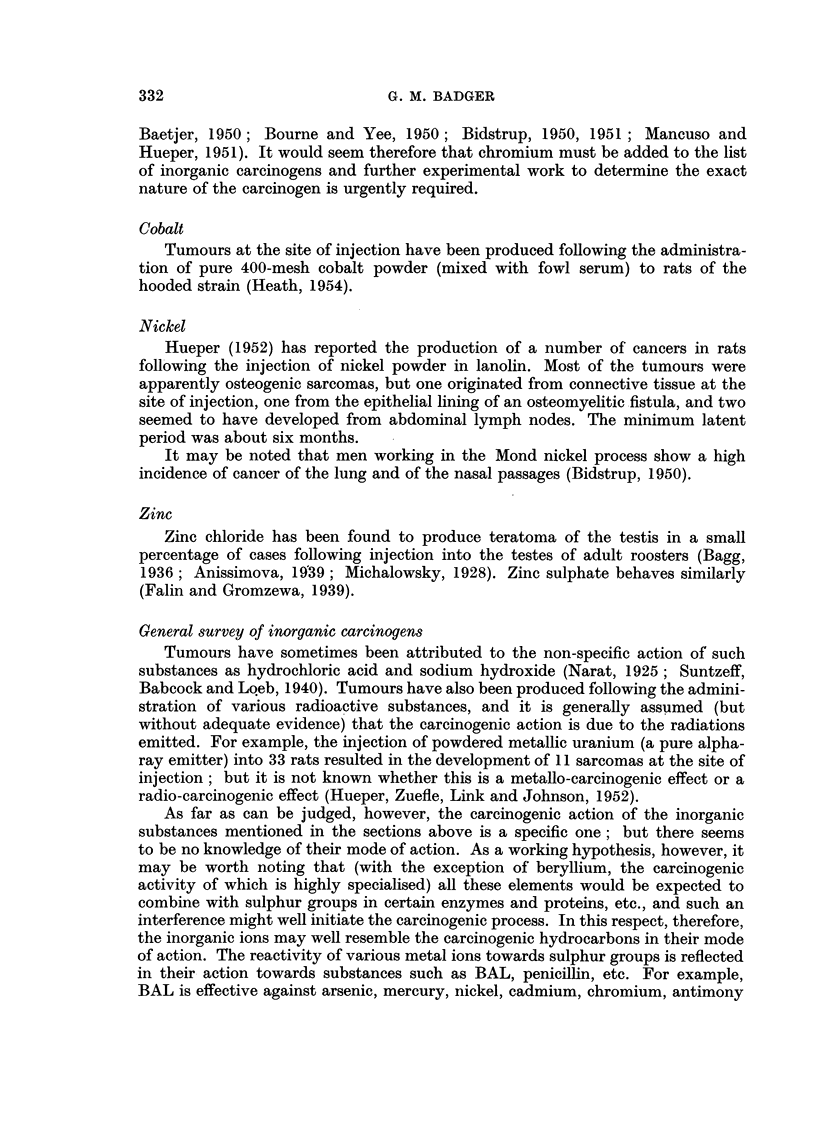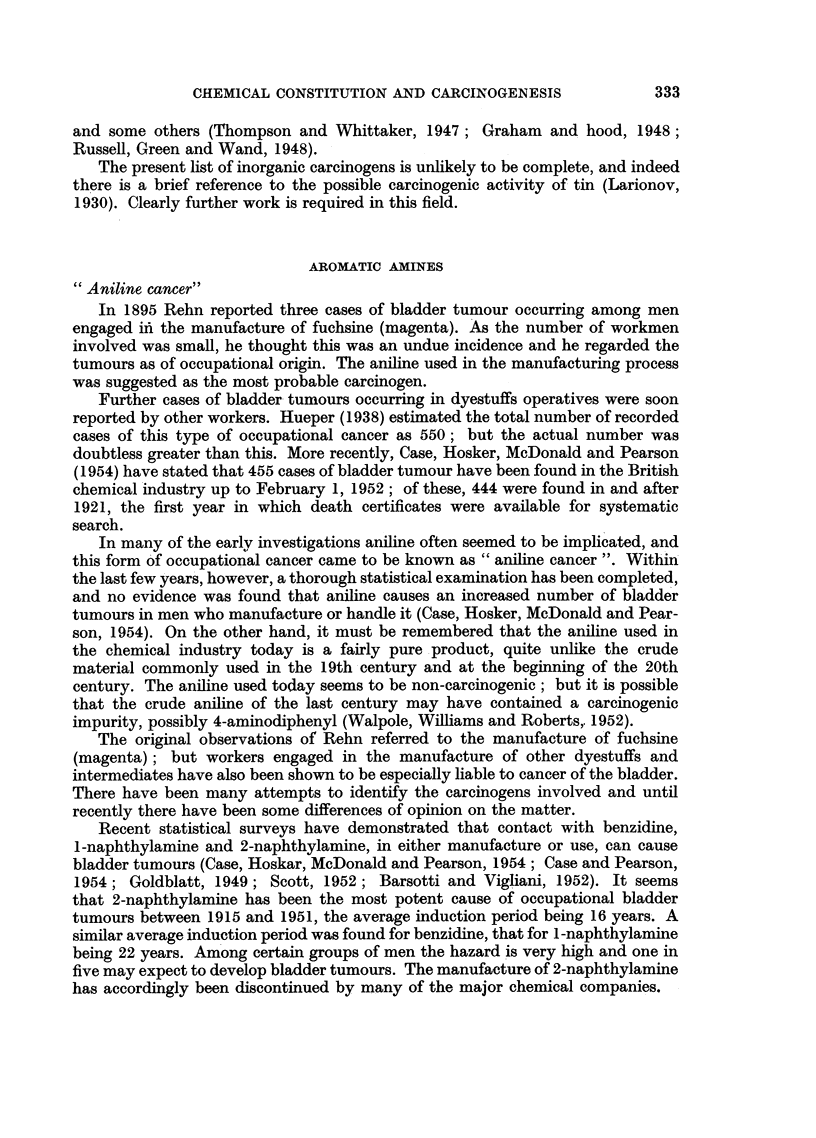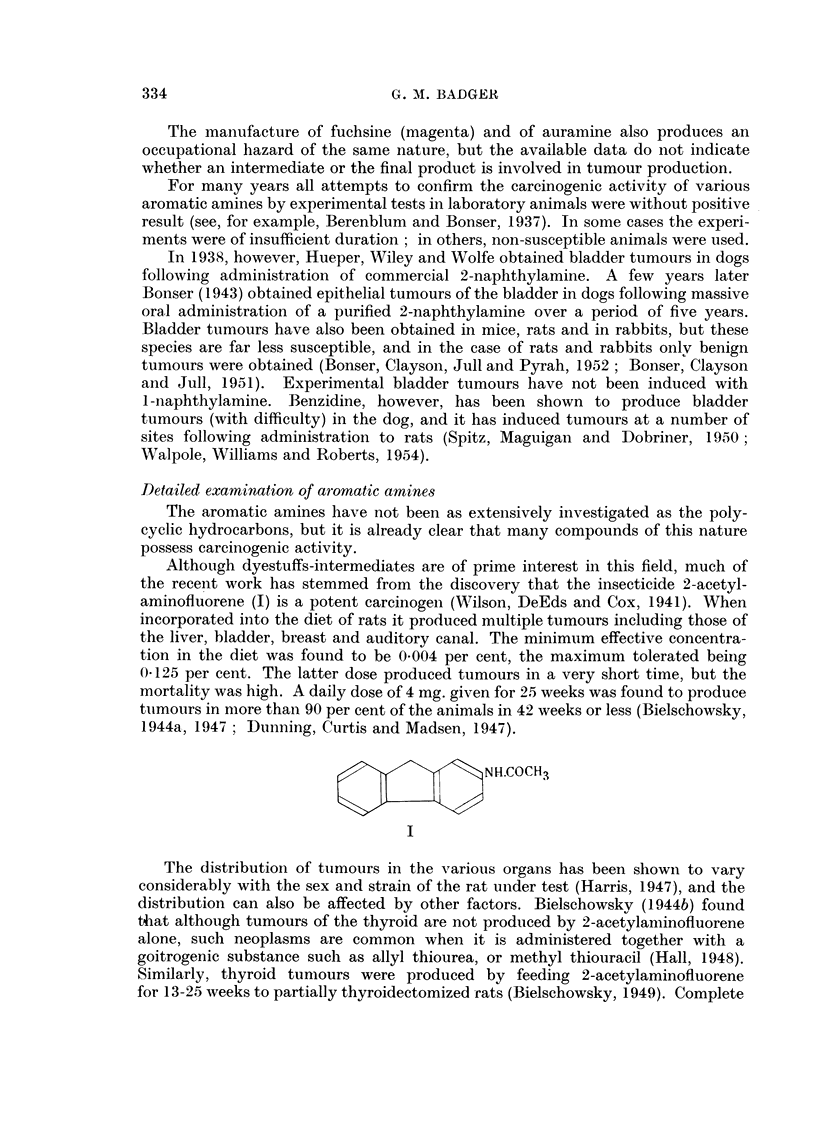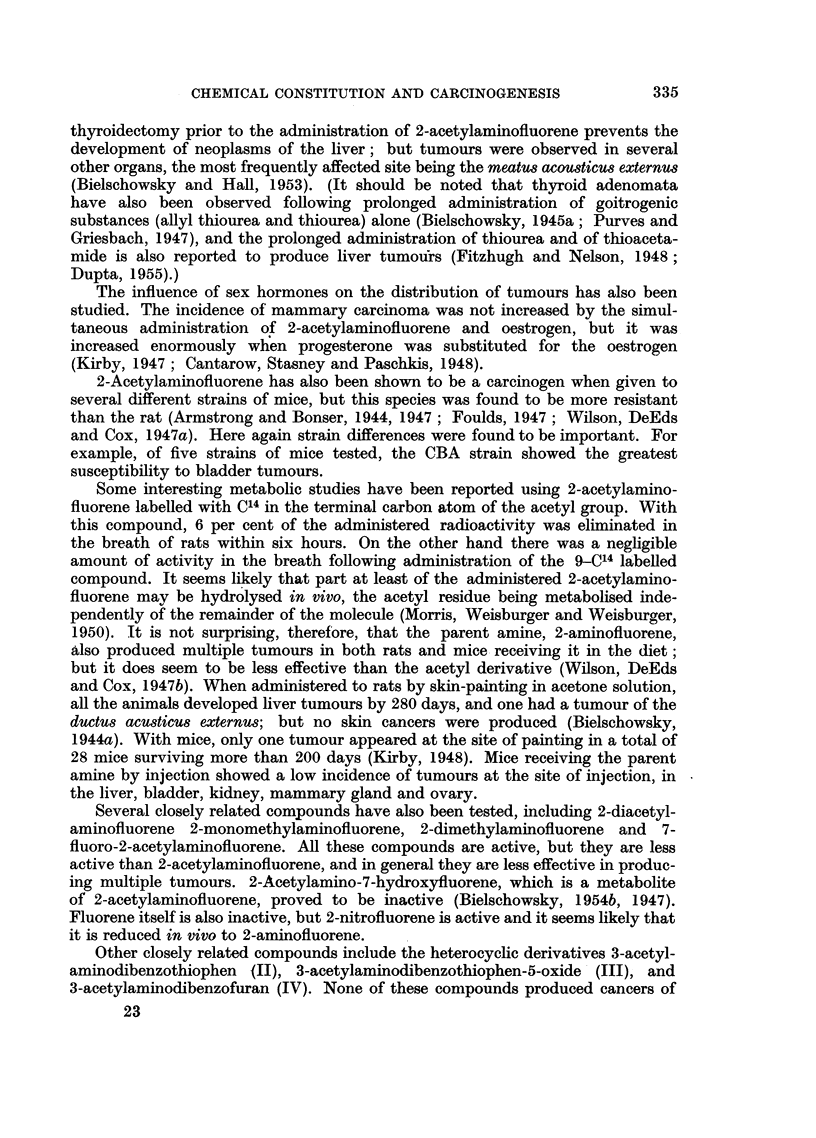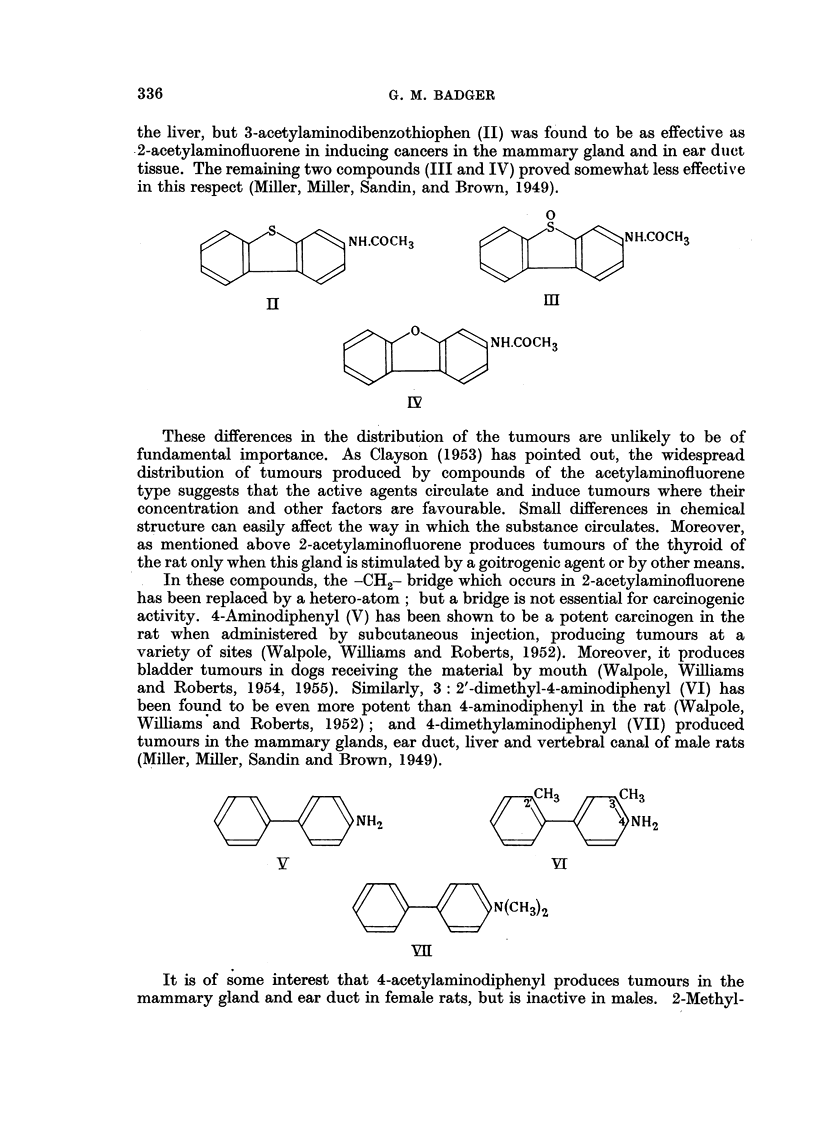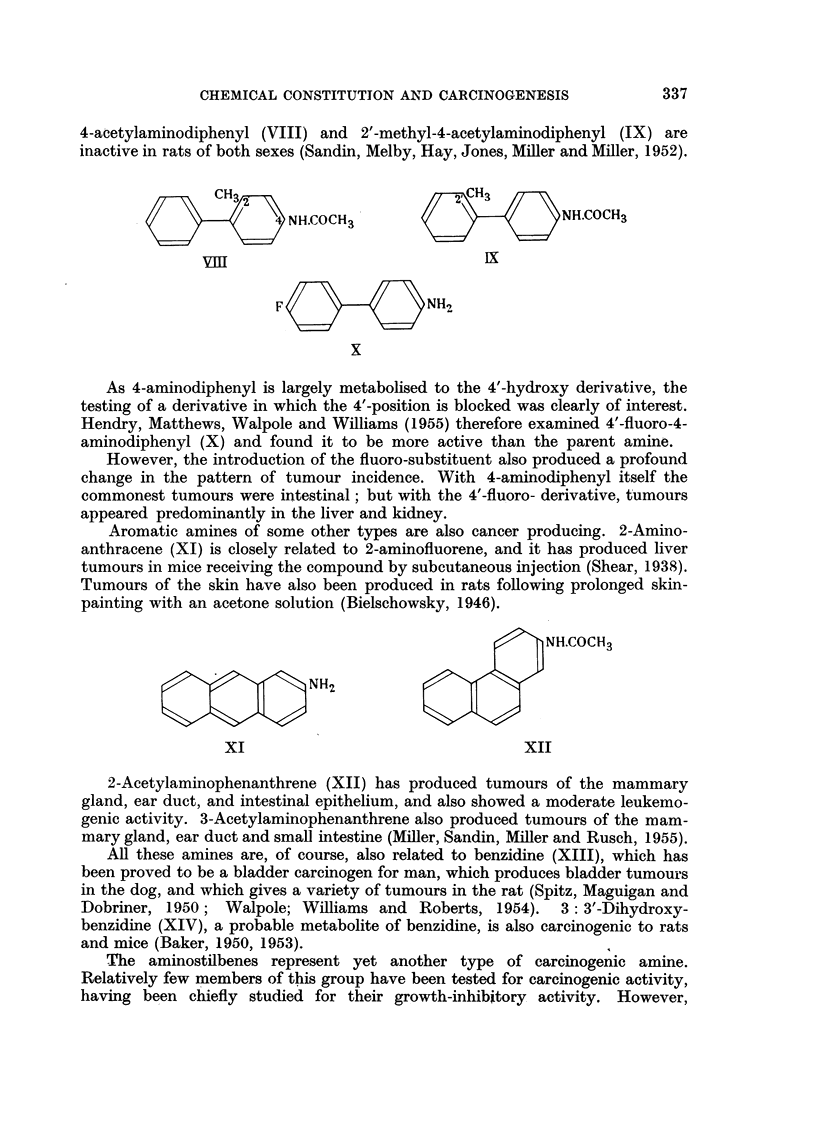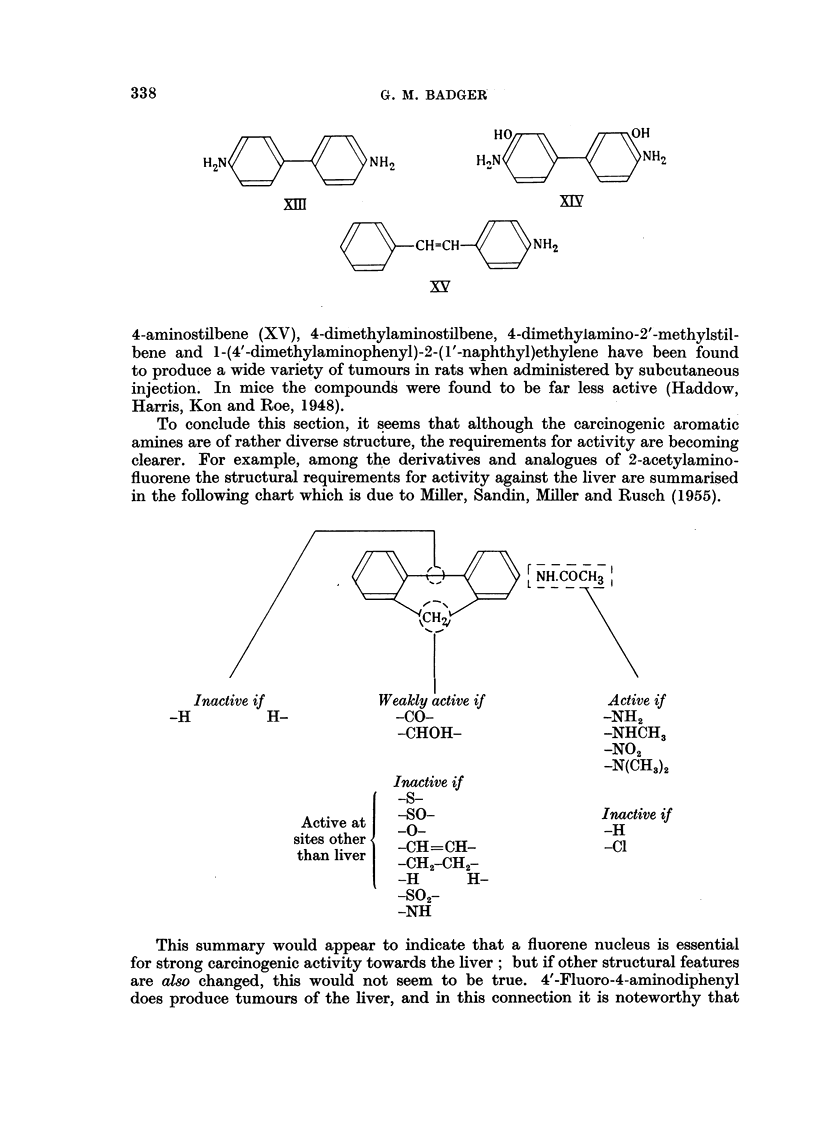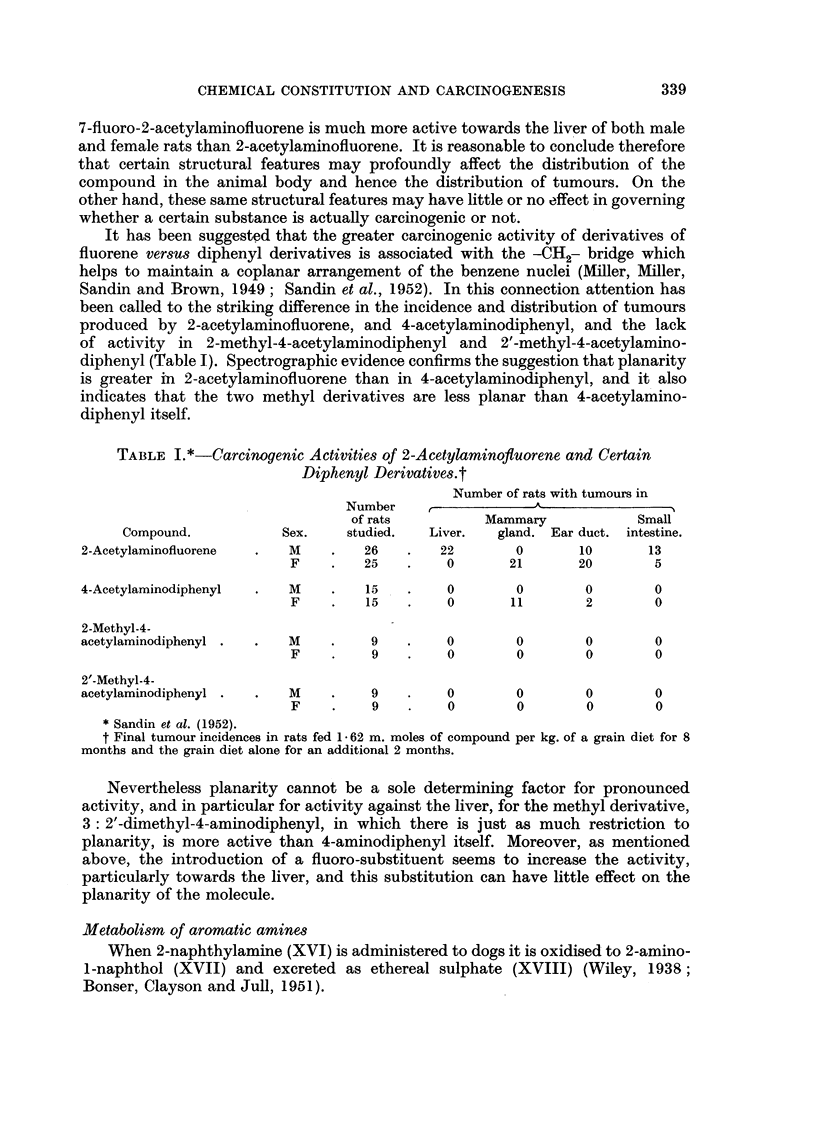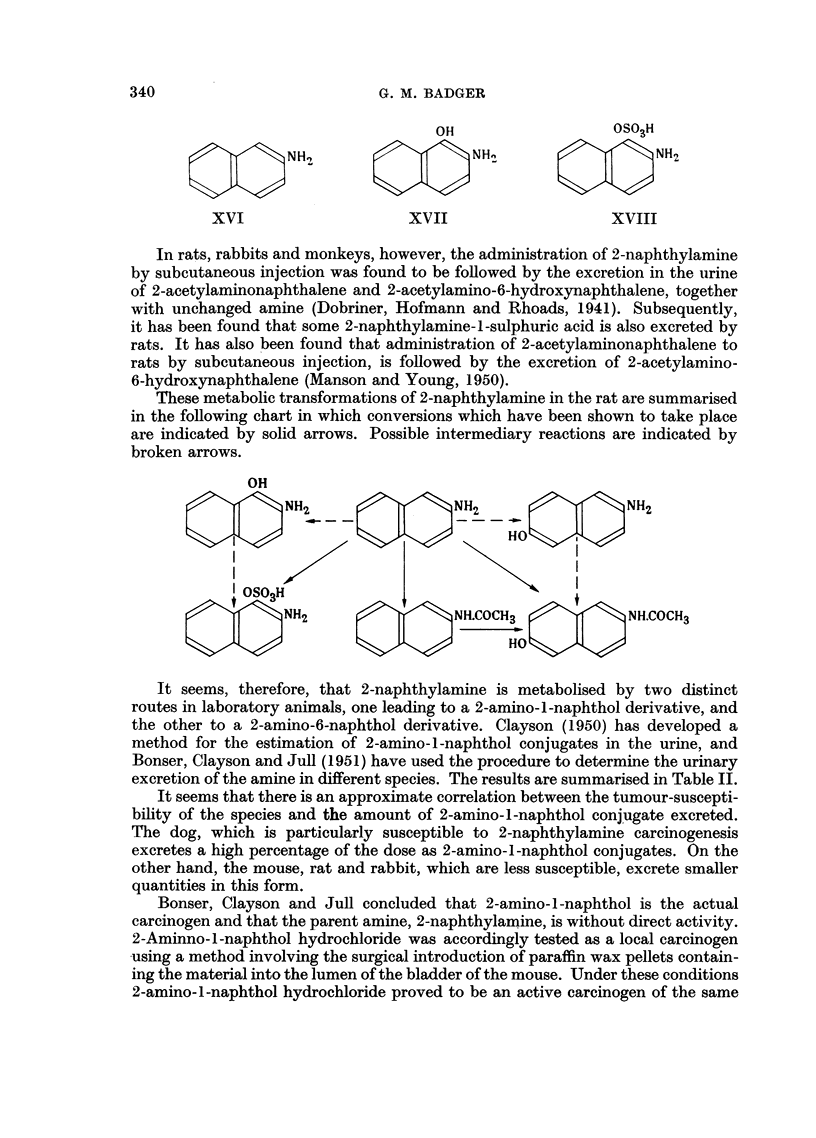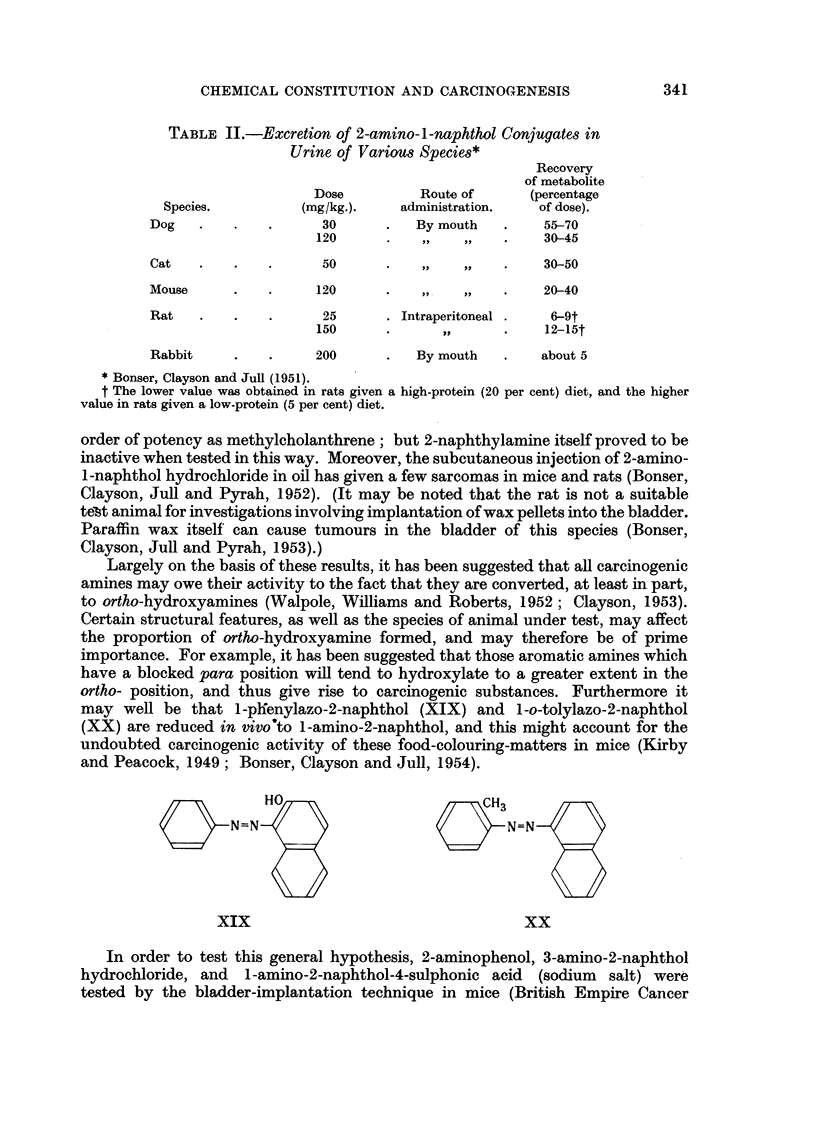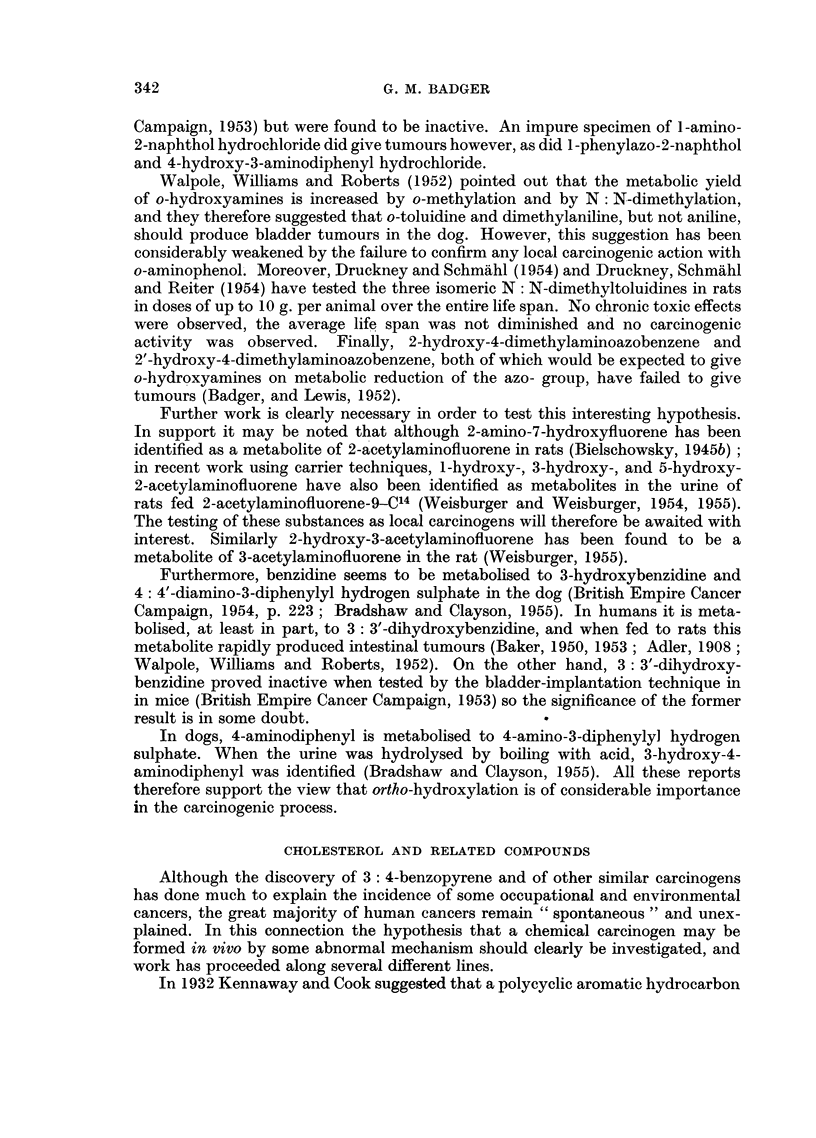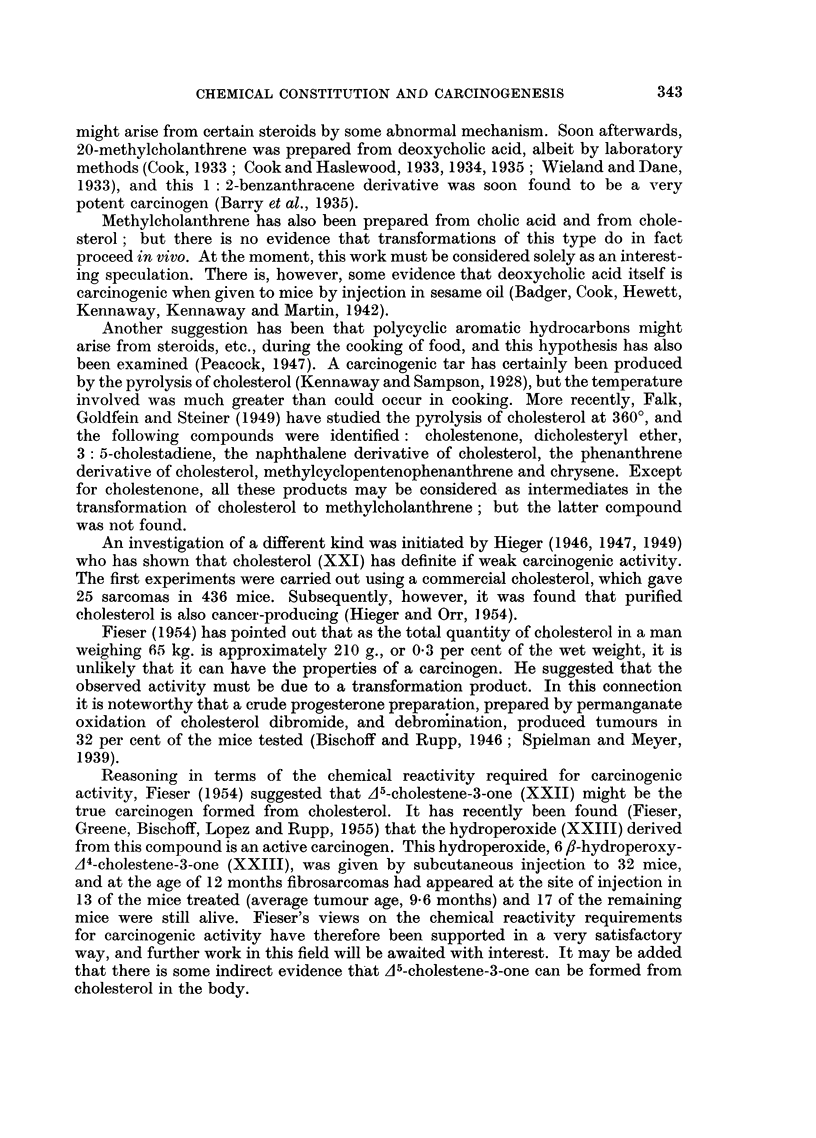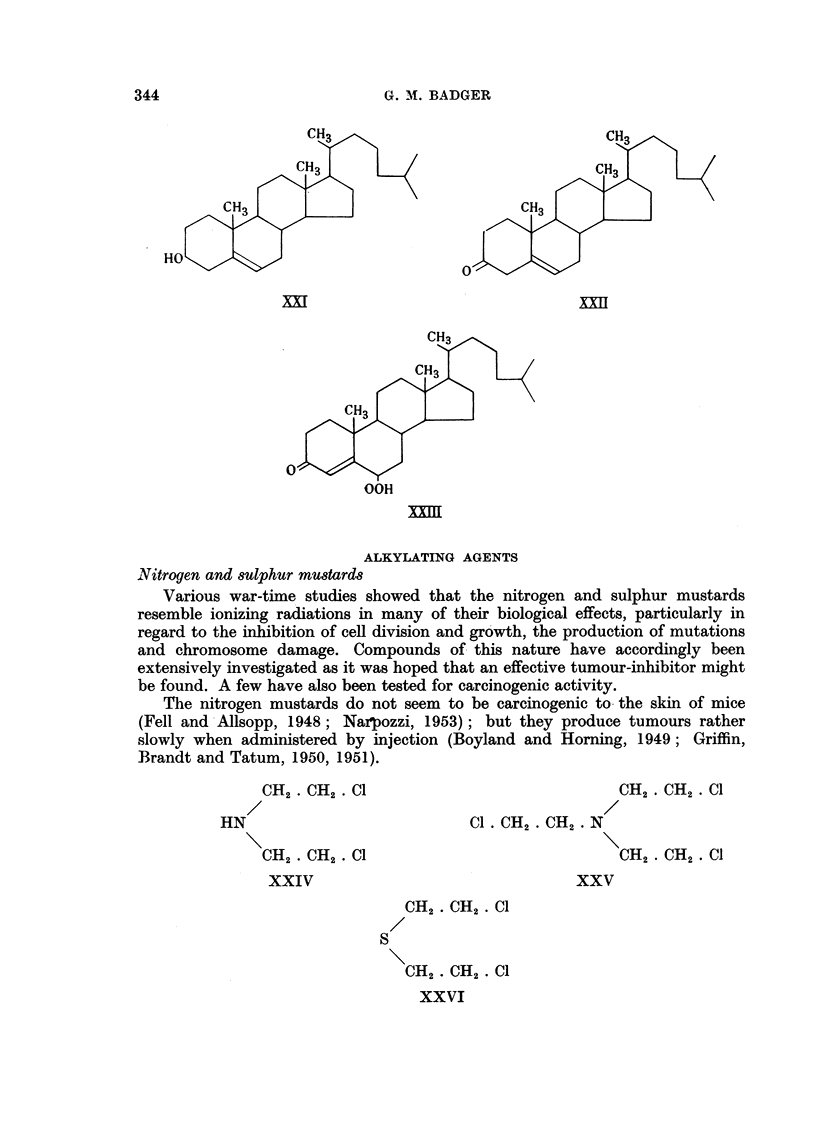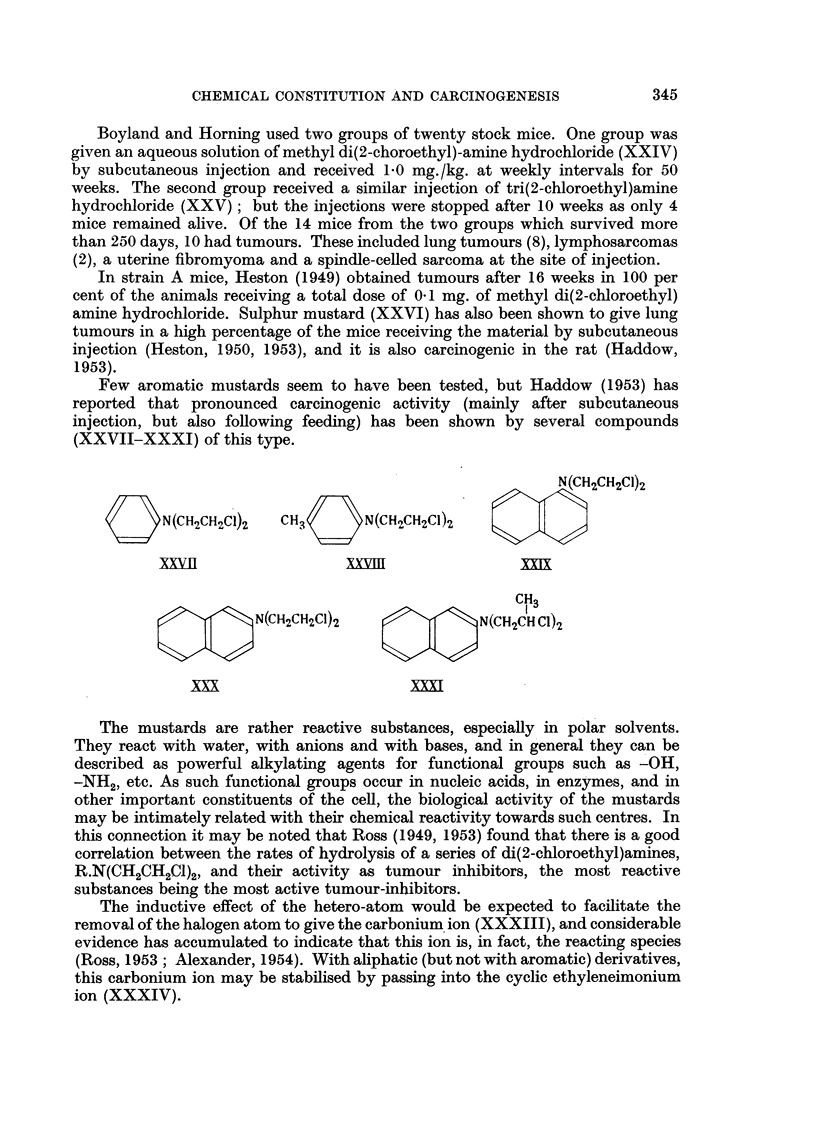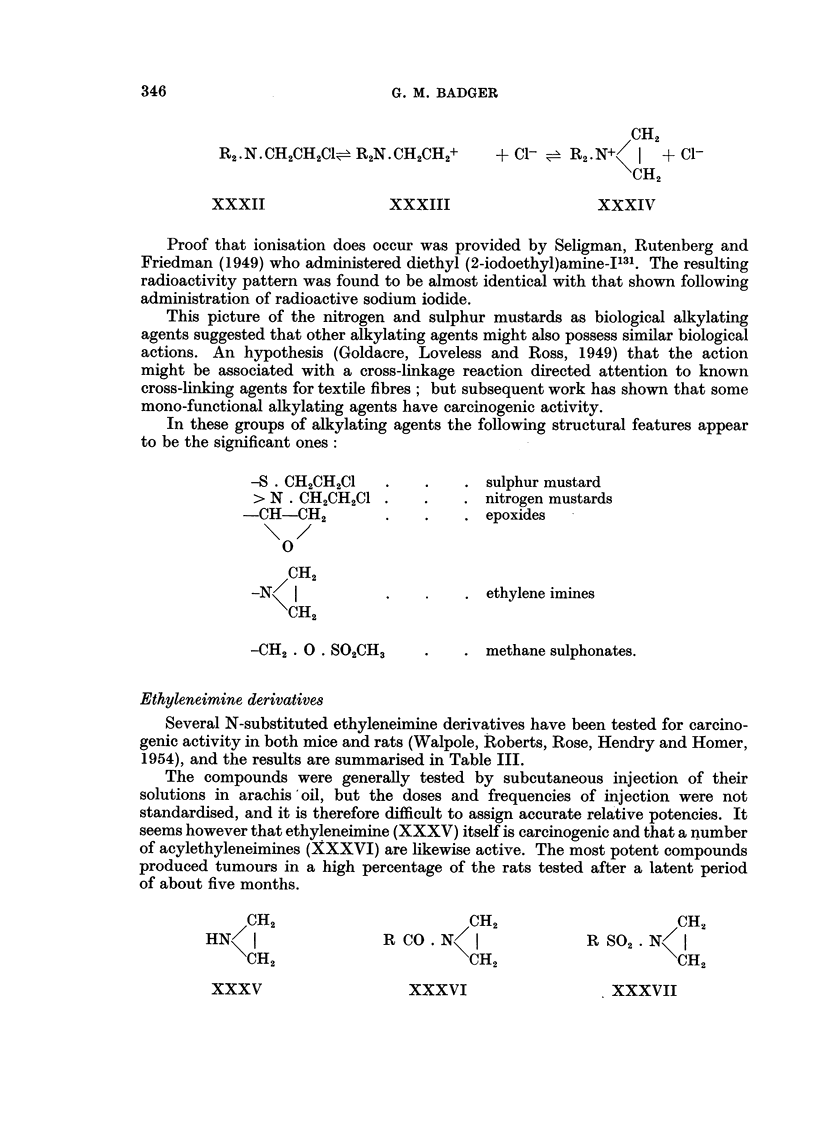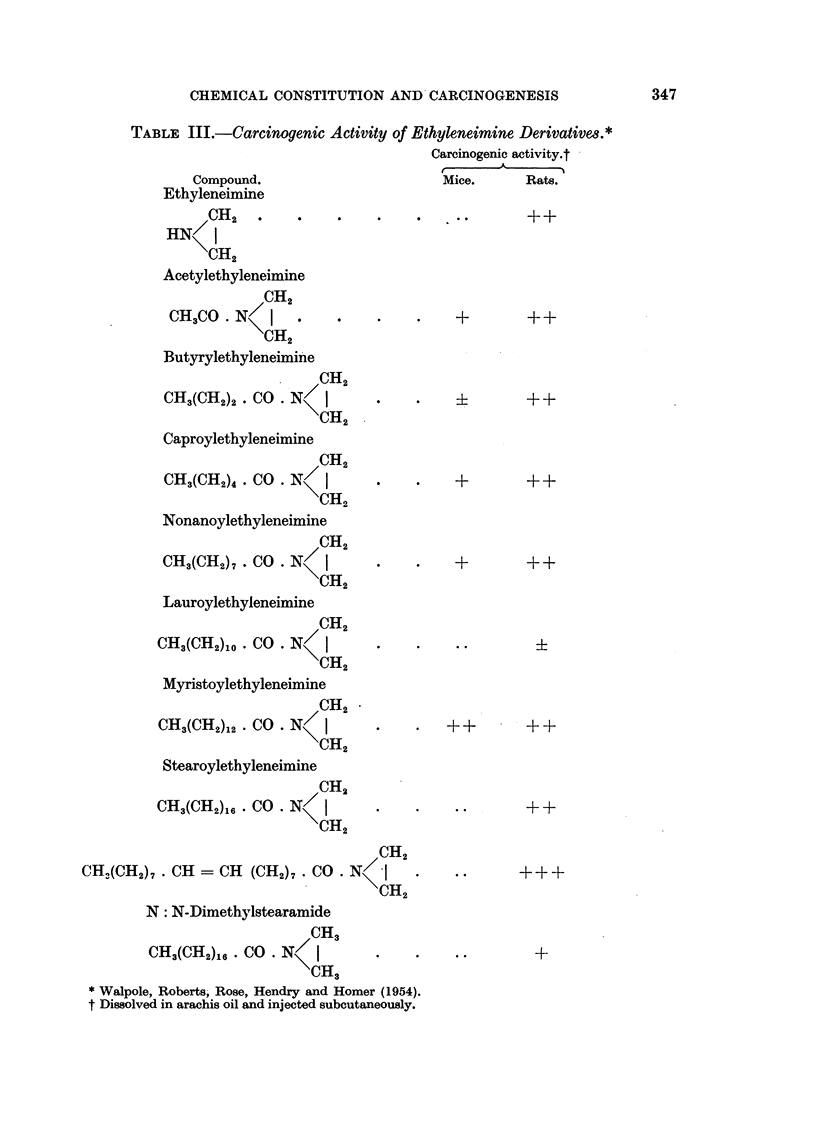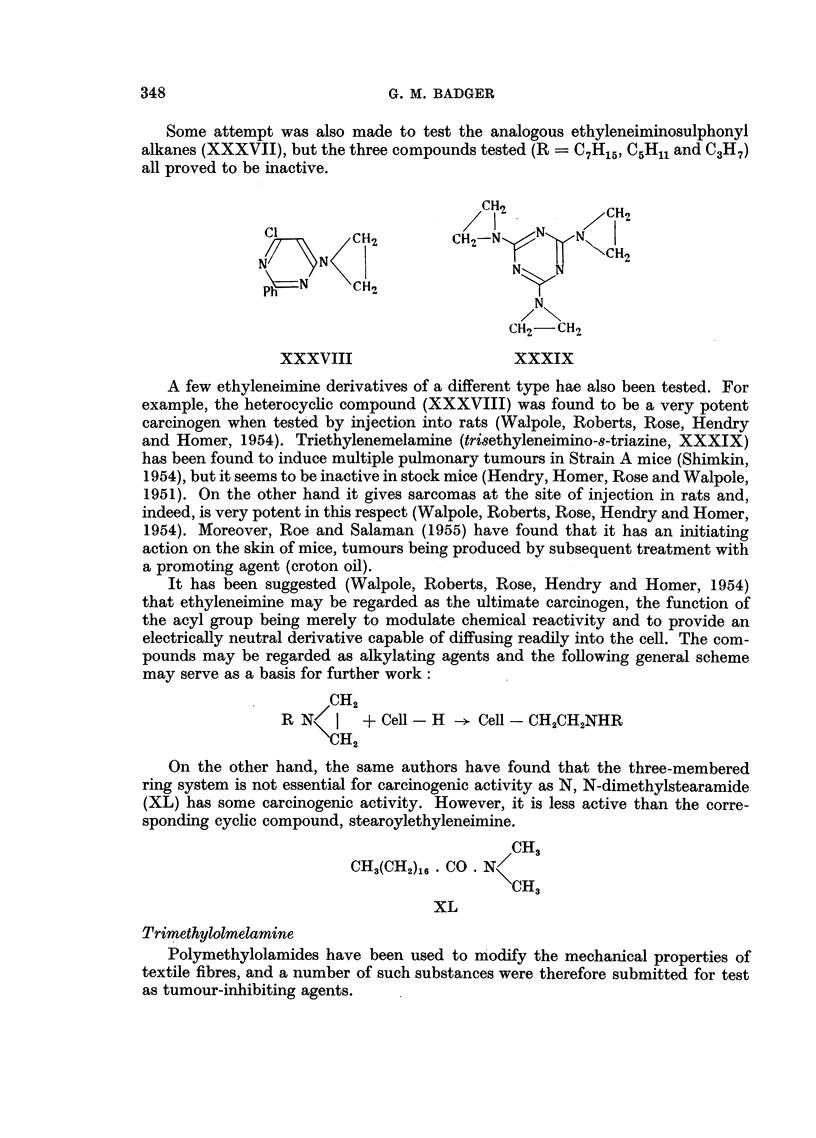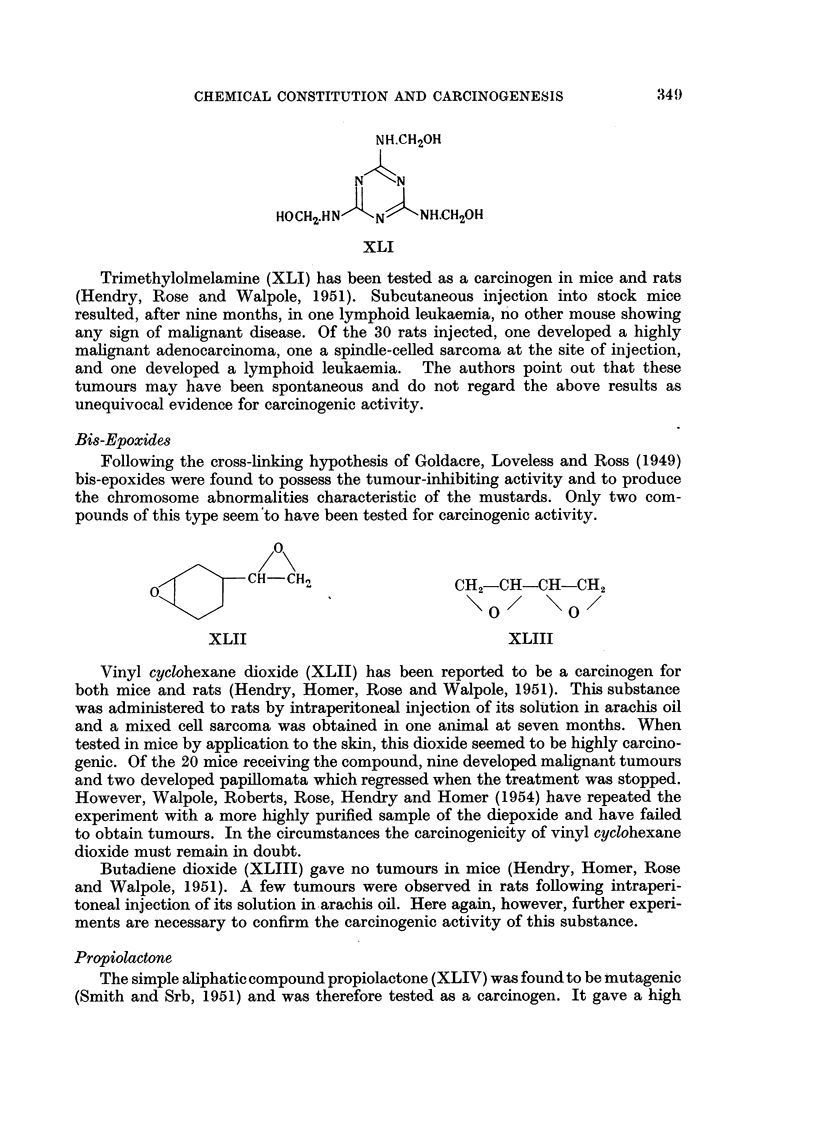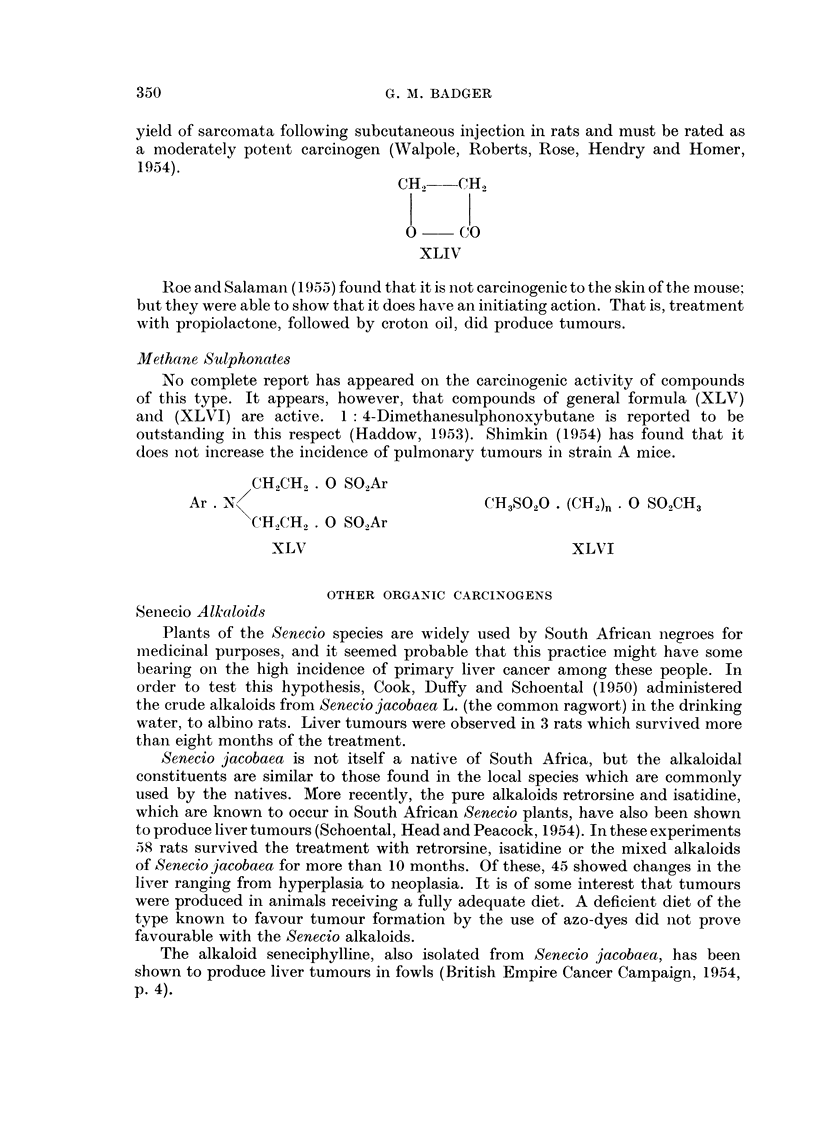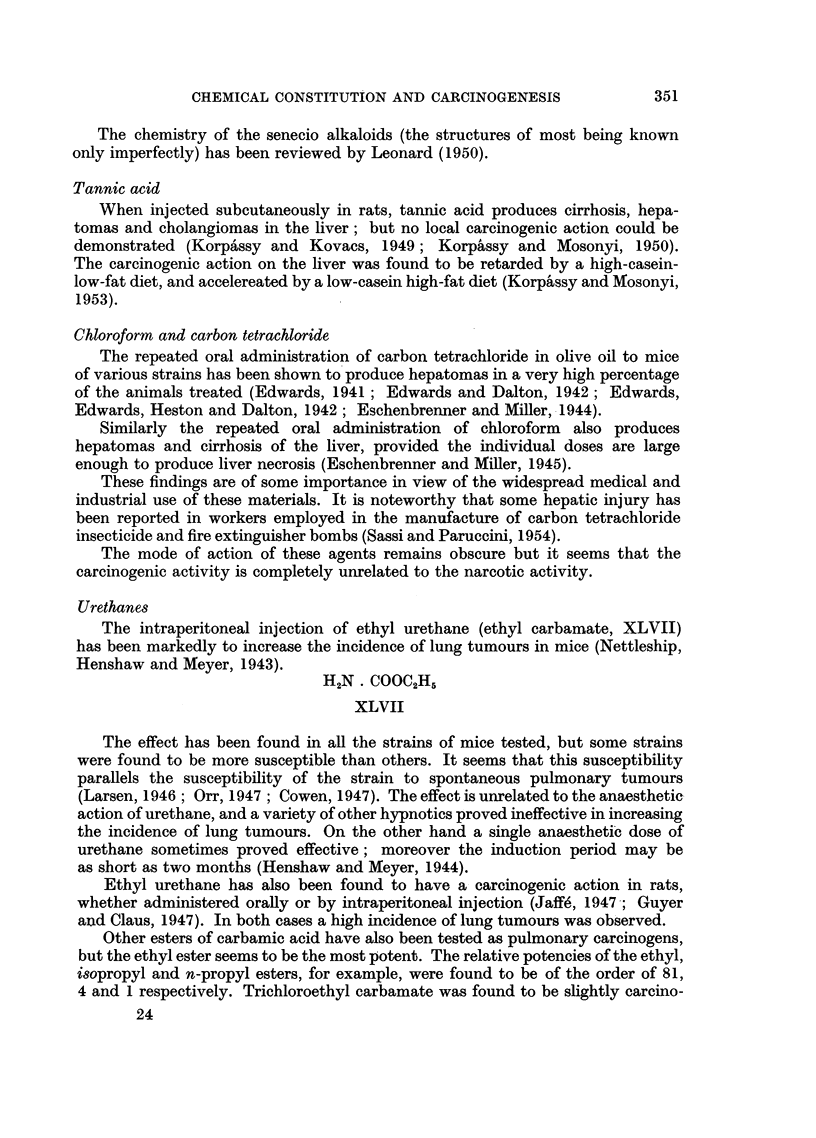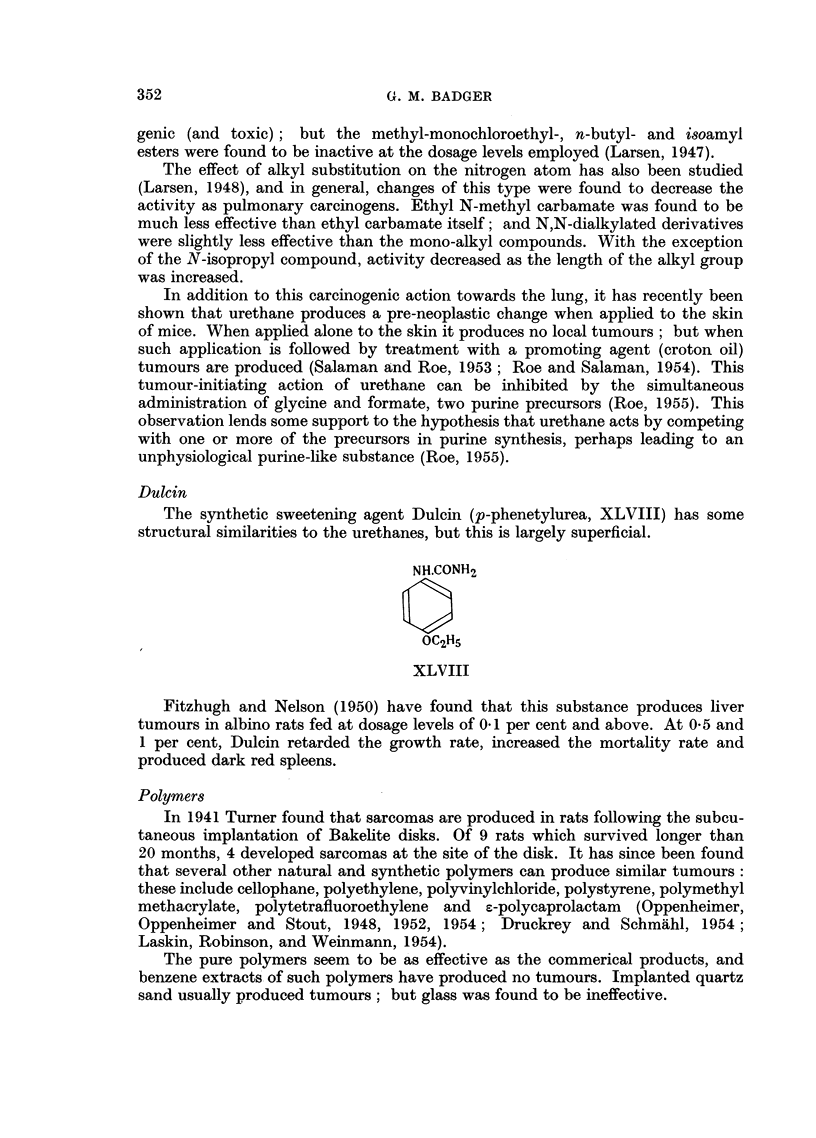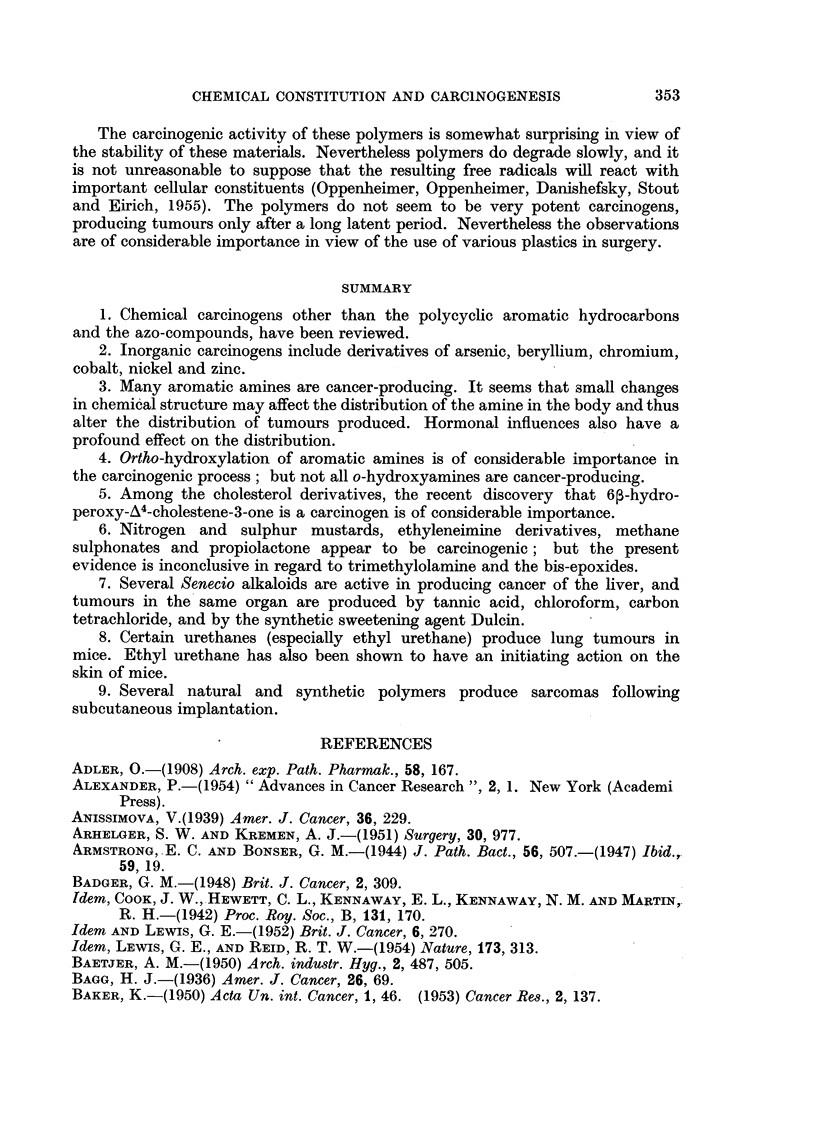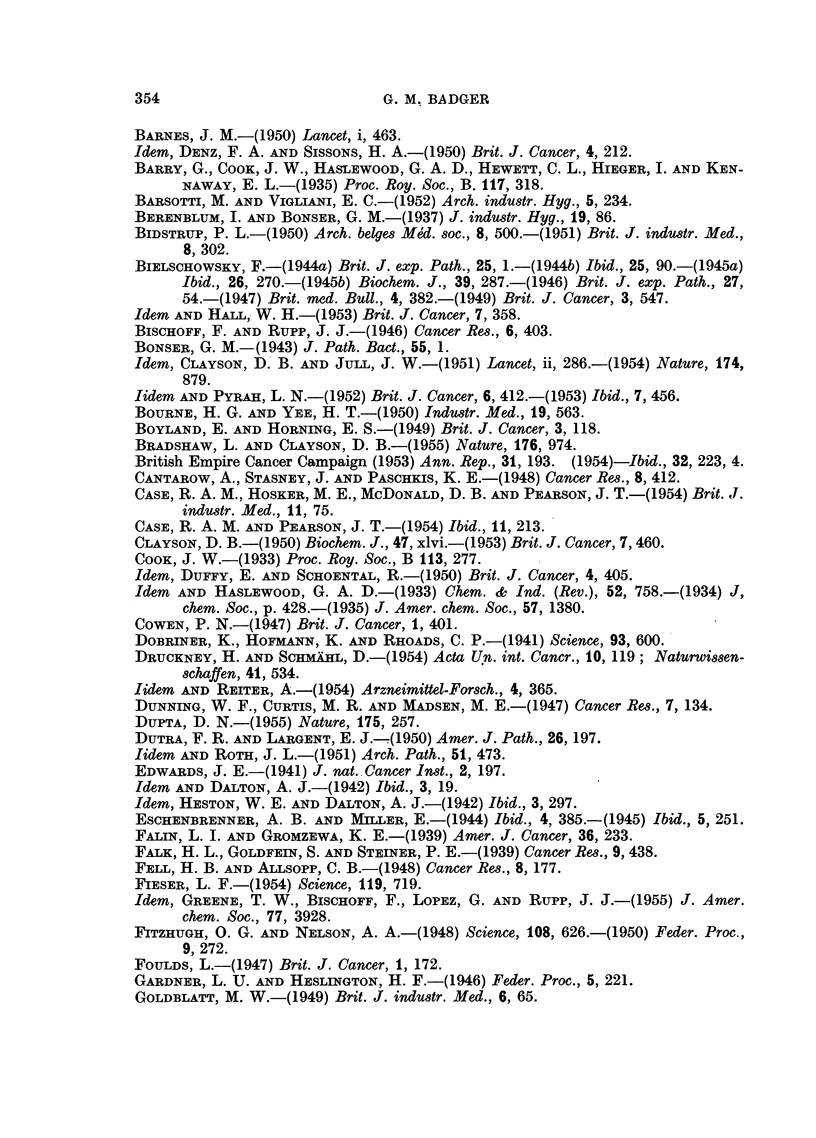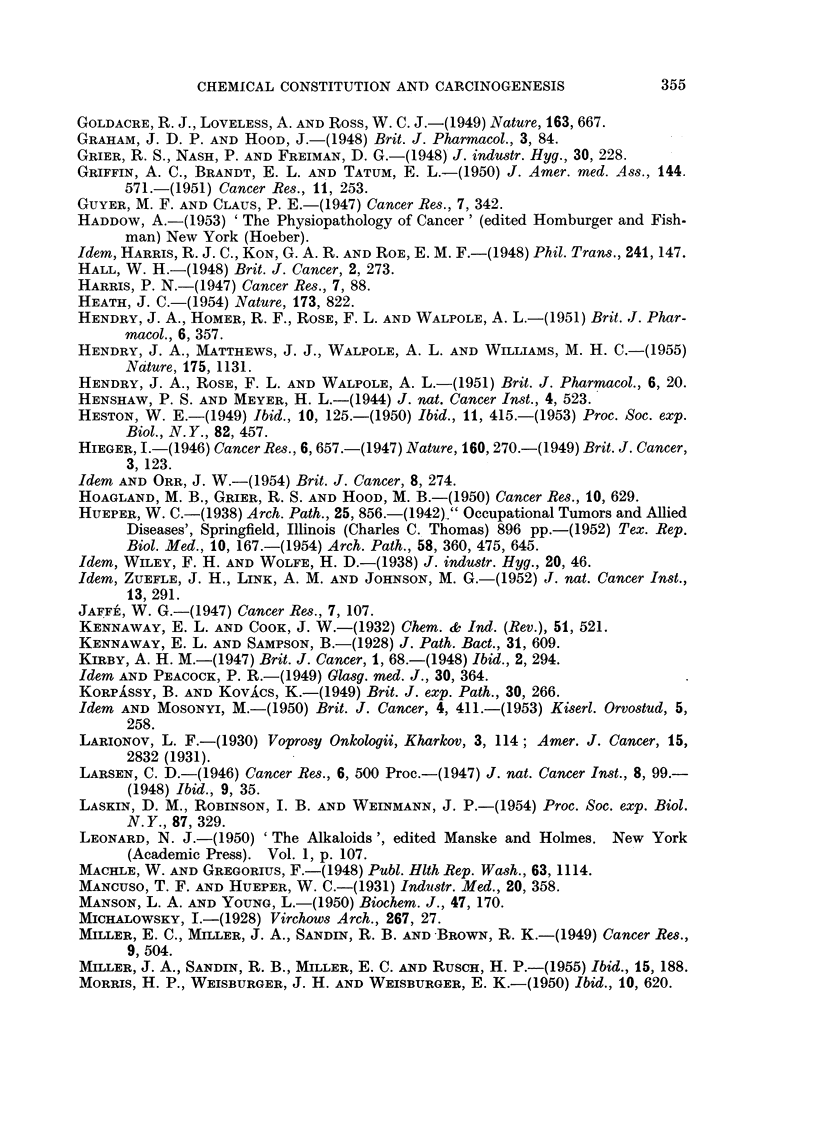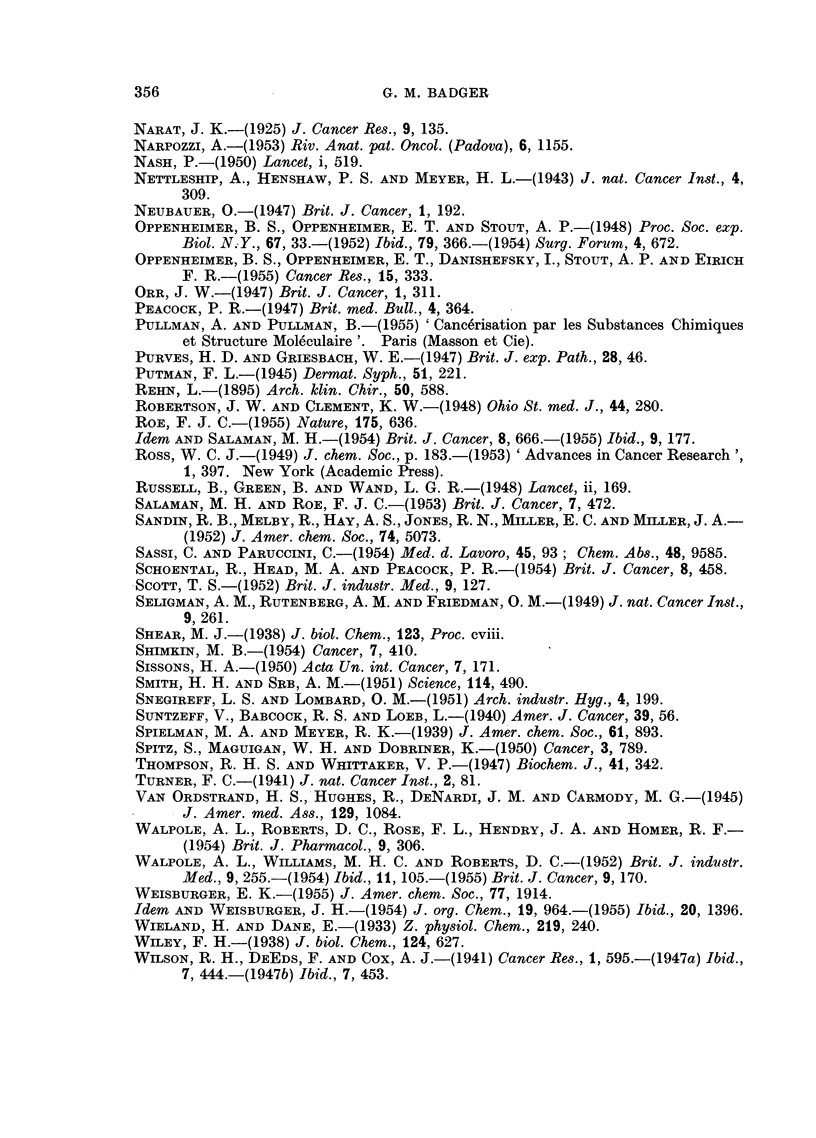# Miscellaneous Chemical Carcinogens: Chemical Constitution and Carcinogenic Activity

**DOI:** 10.1038/bjc.1956.40

**Published:** 1956-06

**Authors:** G. M. Badger


### MISCELLANEOUS CHEMICAL CARCINOGENS: CHEMICAL CONSTITUTION AND CARCINOGENIC ACTIVITY

G. M. BADGER

*From the Organic Chemistry Department, University of Adelaide*

Received for publication March 6, 1956

The carcinogenic hydrocarbons have been very extensively studied from several different points of view. Many problems still await solution, but the relationship between chemical constitution and carcinogenic activity is becoming clearer (Badger, 1948; Pullman and Pullman, 1955). The azo-compounds have also received considerable attention, and here again some relationship between structure and activity is discernible (Badger, and Lewis, 1952; Pullman and Pullman, 1955). Examples of chemical carcinogens belonging to other classes of compound are also known, and in recent years more attention has been devoted to these substances. Certain aromatic amines are carcinogenic as well as some urethanes, chloro-compounds, " mustards ", ethyleneimines, *Senecio* alkaloids, steroids, and some inorganic compounds. The purpose of the present paper is to review the present position with these miscellaneous chemical carcinogens. Radioactive carcinogens and hormonal carcinogens (oestrogens, etc.) are not included.

The diversity of the different types of chemical carcinogen may occasion some surprise. On the other hand, different chemical types are well known to produce many other biological responses. Antibacterial drugs, for example, vary widely in their chemical type, and in their mode of action. In this case it is clearly unprofitable to attempt to find structural relationships between compounds acting by entirely different mechanisms; but the study of the relationship between chemical constitution and antibacterial activity among any one group (say the sulphonamides) may be useful and valuable. In the same way the different types of chemical carcinogens probably have different modes of action. In these circumstances no structural relationship between the polycyclic aromatic hydrocarbons, the ethyleneimines, urethane and carbon tetrachloride, should be looked for. Nevertheless, the study of carcinogenic activity in a series of related aromatic amines may produce information of some importance, as may a similar study of a series of related ethyleneimines, and so on. It should be noted, however, that an observed structural relationship does not necessarily establish that two compounds produce the same biological action by the same mechanism. Most of the sulphonamides probably do have the same mode of action, but in spite of its structural similarity, *p*-sulphonamidobenzylamine is known to differ from sulphanilamide. Furthermore, among the carcinogenic azo-compounds, evidence is accumulating that compounds such as 1-phenylazo-2-naphthol and 2 : 2′-azonaphthalene should be clearly distinguished from the *amino*-azo-compounds such as 4-dimethylaminoazobenzene (Badger, Lewis and Reid, 1954).

### INORGANIC CARCINOGENS

### *Arsenic*

For many years the prolonged exposure to small quantities of arsenic, either in the drinking water, by industrial exposure, or by treatment with arsenical drugs, has been thought to be a contributing factor in certain varieties of cancer (Hueper, 1942, 1954). Much of the early evidence was clearly unsatisfactory, and it is still incomplete, but the general consensus of opinion now seems to be that arsenic must be classed as an exogenous carcinogen having a low order of potency (see however Snegireff and Lombard, 1951). A few cases of occupational arsenical cancer have been noted in factories manufacturing sheep-dip preparations (sodium arsenite, and arsenious acid) and insecticides (copper arsenite, calcium arsenate, or lead arsenate), and a very few in the mining and smelting industry. The literature has been carefully reviewed by Neubauer (1947).

A number of cases of " medicinal " arsenical cancer have also been reported in patients treated with various inorganic arsenical drugs such as potassium arsenite and arsenic trioxide. Neubauer collected 143 such cases from the literature, but more have since been reported (e.g. Arhelger and Kremen, 1951; Robertson and Clement, 1948; Putman, 1945).

The most important trivalent arsenical drugs are the arsphenamines, used for the treatment of syphilis, and Neubauer concludes that these play only an insignificant part in the causation of cancer.

There have been several attempts to produce arsenical cancers in laboratory animals, but no satisfactory evidence of success has been produced. Part of the difficulty is probably associated with the toxic nature of the material; otherwise it must be tentatively assumed that man is more susceptible to arsenic than the laboratory animals used so far.

### *Beryllium*

The production of osteogenic sarcomas in rabbits following the injection of zinc beryllium silicate and beryllium oxide was first noted by Gardner and Heslington (1946), and has since been confirmed by many other workers (Barnes, 1950; Nash, 1950; Hoagland, Grier and Hood, 1950; Dutra and Largent, 1950; Barnes, Denz and Sissons, 1950, Sissons, 1950).

An osteogenic sarcoma has been reported following the inhalation of beryllium oxide (Dutra, Largent and Roth, 1951) and finely powdered beryllium metal has also been shown to be effective when administered by intravenous injection as an aqueous suspension (Barnes, 1950).

This experimental production of tumours using beryllium and beryllium compounds is of considerable importance in view of the industrial use of these materials. Among workers in the industry, pulmonary reactions and skin lesions appear to be common, and subcutaneous granuloma have been reported in persons who cut themselves on fluorescent lamps containing beryllium (Van Ordstrand, Hughes, DeNardi and Carmody, 1945; Grier, Nash and Freiman, 1948).

### *Chromium*

Several workers have reported that the incidence of cancer of the lung is significantly higher among workers in the chromate industry than in comparable groups not exposed to compounds of this metal (Machle and Gregorius, 1948;

Baetjer, 1950; Bourne and Yee, 1950; Bidstrup, 1950, 1951; Mancuso and Hueper, 1951). It would seem therefore that chromium must be added to the list of inorganic carcinogens and further experimental work to determine the exact nature of the carcinogen is urgently required.

*Cobalt*

Tumours at the site of injection have been produced following the administration of pure 400-mesh cobalt powder (mixed with fowl serum) to rats of the hooded strain (Heath, 1954).

*Nickel*

Hueper (1952) has reported the production of a number of cancers in rats following the injection of nickel powder in lanolin. Most of the tumours were apparently osteogenic sarcomas, but one originated from connective tissue at the site of injection, one from the epithelial lining of an osteomyelitic fistula, and two seemed to have developed from abdominal lymph nodes. The minimum latent period was about six months.

It may be noted that men working in the Mond nickel process show a high incidence of cancer of the lung and of the nasal passages (Bidstrup, 1950).

*Zinc*

Zinc chloride has been found to produce teratoma of the testis in a small percentage of cases following injection into the testes of adult roosters (Bagg, 1936; Anissimova, 1939; Michalowsky, 1928). Zinc sulphate behaves similarly (Falin and Gromzewa, 1939).

*General survey of inorganic carcinogens*

Tumours have sometimes been attributed to the non-specific action of such substances as hydrochloric acid and sodium hydroxide (Narat, 1925; Suntzeff, Babcock and Loeb, 1940). Tumours have also been produced following the administration of various radioactive substances, and it is generally assumed (but without adequate evidence) that the carcinogenic action is due to the radiations emitted. For example, the injection of powdered metallic uranium (a pure alpha-ray emitter) into 33 rats resulted in the development of 11 sarcomas at the site of injection; but it is not known whether this is a metallo-carcinogenic effect or a radio-carcinogenic effect (Hueper, Zuefle, Link and Johnson, 1952).

As far as can be judged, however, the carcinogenic action of the inorganic substances mentioned in the sections above is a specific one; but there seems to be no knowledge of their mode of action. As a working hypothesis, however, it may be worth noting that (with the exception of beryllium, the carcinogenic activity of which is highly specialised) all these elements would be expected to combine with sulphur groups in certain enzymes and proteins, etc., and such an interference might well initiate the carcinogenic process. In this respect, therefore, the inorganic ions may well resemble the carcinogenic hydrocarbons in their mode of action. The reactivity of various metal ions towards sulphur groups is reflected in their action towards substances such as BAL, penicillin, etc. For example, BAL is effective against arsenic, mercury, nickel, cadmium, chromium, antimony and some others (Thompson and Whittaker, 1947 ; Graham and hood, 1948 ; Russell, Green and Wand, 1948).

The present list of inorganic carcinogens is unlikely to be complete, and indeed there is a brief reference to the possible carcinogenic activity of tin (Larionov, 1930). Clearly further work is required in this field.

### AROMATIC AMINES

*" Aniline cancer"*

In 1895 Rehn reported three cases of bladder tumour occurring among men engaged in the manufacture of fuchsine (magenta). As the number of workmen involved was small, he thought this was an undue incidence and he regarded the tumours as of occupational origin. The aniline used in the manufacturing process was suggested as the most probable carcinogen.

Further cases of bladder tumours occurring in dyestuffs operatives were soon reported by other workers. Hueper (1938) estimated the total number of recorded cases of this type of occupational cancer as 550 ; but the actual number was doubtless greater than this. More recently, Case, Hosker, McDonald and Pearson (1954) have stated that 455 cases of bladder tumour have been found in the British chemical industry up to February 1, 1952 ; of these, 444 were found in and after 1921, the first year in which death certificates were available for systematic search.

In many of the early investigations aniline often seemed to be implicated, and this form of occupational cancer came to be known as " aniline cancer ". Within the last few years, however, a thorough statistical examination has been completed, and no evidence was found that aniline causes an increased number of bladder tumours in men who manufacture or handle it (Case, Hosker, McDonald and Pearson, 1954). On the other hand, it must be remembered that the aniline used in the chemical industry today is a fairly pure product, quite unlike the crude material commonly used in the 19th century and at the beginning of the 20th century. The aniline used today seems to be non-carcinogenic ; but it is possible that the crude aniline of the last century may have contained a carcinogenic impurity, possibly 4-aminodiphenyl (Walpole, Williams and Roberts, 1952).

The original observations of Rehn referred to the manufacture of fuchsine (magenta) ; but workers engaged in the manufacture of other dyestuffs and intermediates have also been shown to be especially liable to cancer of the bladder. There have been many attempts to identify the carcinogens involved and until recently there have been some differences of opinion on the matter.

Recent statistical surveys have demonstrated that contact with benzidine, 1-naphthylamine and 2-naphthylamine, in either manufacture or use, can cause bladder tumours (Case, Hoskar, McDonald and Pearson, 1954 ; Case and Pearson, 1954 ; Goldblatt, 1949 ; Scott, 1952 ; Barsotti and Vigliani, 1952). It seems that 2-naphthylamine has been the most potent cause of occupational bladder tumours between 1915 and 1951, the average induction period being 16 years. A similar average induction period was found for benzidine, that for 1-naphthylamine being 22 years. Among certain groups of men the hazard is very high and one in five may expect to develop bladder tumours. The manufacture of 2-naphthylamine has accordingly been discontinued by many of the major chemical companies.

The manufacture of fuchsine (magenta) and of auramine also produces an occupational hazard of the same nature, but the available data do not indicate whether an intermediate or the final product is involved in tumour production.

For many years all attempts to confirm the carcinogenic activity of various aromatic amines by experimental tests in laboratory animals were without positive result (see, for example, Berenblum and Bonser, 1937). In some cases the experiments were of insufficient duration ; in others, non-susceptible animals were used.

In 1938, however, Hueper, Wiley and Wolfe obtained bladder tumours in dogs following administration of commercial 2-naphthylamine. A few years later Bonser (1943) obtained epithelial tumours of the bladder in dogs following massive oral administration of a purified 2-naphthylamine over a period of five years. Bladder tumours have also been obtained in mice, rats and in rabbits, but these species are far less susceptible, and in the case of rats and rabbits only benign tumours were obtained (Bonser, Clayson, Jull and Pyrah, 1952 ; Bonser, Clayson and Jull, 1951). Experimental bladder tumours have not been induced with 1-naphthylamine. Benzidine, however, has been shown to produce bladder tumours (with difficulty) in the dog, and it has induced tumours at a number of sites following administration to rats (Spitz, Maguigan and Dobriner, 1950 ; Walpole, Williams and Roberts, 1954).

*Detailed examination of aromatic amines*

The aromatic amines have not been as extensively investigated as the polycyclic hydrocarbons, but it is already clear that many compounds of this nature possess carcinogenic activity.

Although dyestuffs-intermediates are of prime interest in this field, much of the recent work has stemmed from the discovery that the insecticide 2-acetylaminofluorene (I) is a potent carcinogen (Wilson, DeEds and Cox, 1941). When incorporated into the diet of rats it produced multiple tumours including those of the liver, bladder, breast and auditory canal. The minimum effective concentration in the diet was found to be 0·004 per cent, the maximum tolerated being 0·125 per cent. The latter dose produced tumours in a very short time, but the mortality was high. A daily dose of 4 mg. given for 25 weeks was found to produce tumours in more than 90 per cent of the animals in 42 weeks or less (Bielschowsky, 1944a, 1947 ; Dunning, Curtis and Madsen, 1947).

I

The distribution of tumours in the various organs has been shown to vary considerably with the sex and strain of the rat under test (Harris, 1947), and the distribution can also be affected by other factors. Bielschowsky (1944*b*) found that although tumours of the thyroid are not produced by 2-acetylaminofluorene alone, such neoplasms are common when it is administered together with a goitrogenic substance such as allyl thiourea, or methyl thiouracil (Hall, 1948). Similarly, thyroid tumours were produced by feeding 2-acetylaminofluorene for 13-25 weeks to partially thyroidectomized rats (Bielschowsky, 1949). Complete thyroidectomy prior to the administration of 2-acetylaminofluorene prevents the development of neoplasms of the liver; but tumours were observed in several other organs, the most frequently affected site being the *meatus acousticus externus* (Bielschowsky and Hall, 1953). (It should be noted that thyroid adenomata have also been observed following prolonged administration of goitrogenic substances (allyl thiourea and thiourea) alone (Bielschowsky, 1945a; Purves and Griesbach, 1947), and the prolonged administration of thiourea and of thioacetamide is also reported to produce liver tumours (Fitzhugh and Nelson, 1948; Dupta, 1955).)

The influence of sex hormones on the distribution of tumours has also been studied. The incidence of mammary carcinoma was not increased by the simultaneous administration of 2-acetylaminofluorene and oestrogen, but it was increased enormously when progesterone was substituted for the oestrogen (Kirby, 1947; Cantarow, Stasney and Paschkis, 1948).

2-Acetylaminofluorene has also been shown to be a carcinogen when given to several different strains of mice, but this species was found to be more resistant than the rat (Armstrong and Bonser, 1944, 1947; Foulds, 1947; Wilson, DeEds and Cox, 1947*a*). Here again strain differences were found to be important. For example, of five strains of mice tested, the CBA strain showed the greatest susceptibility to bladder tumours.

Some interesting metabolic studies have been reported using 2-acetylaminofluorene labelled with $C^{14}$ in the terminal carbon atom of the acetyl group. With this compound, 6 per cent of the administered radioactivity was eliminated in the breath of rats within six hours. On the other hand there was a negligible amount of activity in the breath following administration of the 9–$C^{14}$ labelled compound. It seems likely that part at least of the administered 2-acetylaminofluorene may be hydrolysed *in vivo*, the acetyl residue being metabolised independently of the remainder of the molecule (Morris, Weisburger and Weisburger, 1950). It is not surprising, therefore, that the parent amine, 2-aminofluorene, also produced multiple tumours in both rats and mice receiving it in the diet; but it does seem to be less effective than the acetyl derivative (Wilson, DeEds and Cox, 1947*b*). When administered to rats by skin-painting in acetone solution, all the animals developed liver tumours by 280 days, and one had a tumour of the *ductus acusticus externus*; but no skin cancers were produced (Bielschowsky, 1944*a*). With mice, only one tumour appeared at the site of painting in a total of 28 mice surviving more than 200 days (Kirby, 1948). Mice receiving the parent amine by injection showed a low incidence of tumours at the site of injection, in the liver, bladder, kidney, mammary gland and ovary.

Several closely related compounds have also been tested, including 2-diacetylaminofluorene 2-monomethylaminofluorene, 2-dimethylaminofluorene and 7-fluoro-2-acetylaminofluorene. All these compounds are active, but they are less active than 2-acetylaminofluorene, and in general they are less effective in producing multiple tumours. 2-Acetylamino-7-hydroxyfluorene, which is a metabolite of 2-acetylaminofluorene, proved to be inactive (Bielschowsky, 1954*b*, 1947). Fluorene itself is also inactive, but 2-nitrofluorene is active and it seems likely that it is reduced *in vivo* to 2-aminofluorene.

Other closely related compounds include the heterocyclic derivatives 3-acetylaminodibenzothiophen (II), 3-acetylaminodibenzothiophen-5-oxide (III), and 3-acetylaminodibenzofuran (IV). None of these compounds produced cancers of the liver, but 3-acetylaminodibenzothiophen (II) was found to be as effective as 2-acetylaminofluorene in inducing cancers in the mammary gland and in ear duct tissue. The remaining two compounds (III and IV) proved somewhat less effective in this respect (Miller, Miller, Sandin, and Brown, 1949).

II

III

IV

These differences in the distribution of the tumours are unlikely to be of fundamental importance. As Clayson (1953) has pointed out, the widespread distribution of tumours produced by compounds of the acetylaminofluorene type suggests that the active agents circulate and induce tumours where their concentration and other factors are favourable. Small differences in chemical structure can easily affect the way in which the substance circulates. Moreover, as mentioned above 2-acetylaminofluorene produces tumours of the thyroid of the rat only when this gland is stimulated by a goitrogenic agent or by other means.

In these compounds, the $-CH_2-$ bridge which occurs in 2-acetylaminofluorene has been replaced by a hetero-atom ; but a bridge is not essential for carcinogenic activity. 4-Aminodiphenyl (V) has been shown to be a potent carcinogen in the rat when administered by subcutaneous injection, producing tumours at a variety of sites (Walpole, Williams and Roberts, 1952). Moreover, it produces bladder tumours in dogs receiving the material by mouth (Walpole, Williams and Roberts, 1954, 1955). Similarly, 3 : 2′-dimethyl-4-aminodiphenyl (VI) has been found to be even more potent than 4-aminodiphenyl in the rat (Walpole, Williams and Roberts, 1952) ; and 4-dimethylaminodiphenyl (VII) produced tumours in the mammary glands, ear duct, liver and vertebral canal of male rats (Miller, Miller, Sandin and Brown, 1949).

V

VI

VII

It is of some interest that 4-acetylaminodiphenyl produces tumours in the mammary gland and ear duct in female rats, but is inactive in males. 2-Methyl-

4-acetylaminodiphenyl (VIII) and 2′-methyl-4-acetylaminodiphenyl (IX) are inactive in rats of both sexes (Sandin, Melby, Hay, Jones, Miller and Miller, 1952).

VIII

IX

X

As 4-aminodiphenyl is largely metabolised to the 4′-hydroxy derivative, the testing of a derivative in which the 4′-position is blocked was clearly of interest. Hendry, Matthews, Walpole and Williams (1955) therefore examined 4′-fluoro-4-aminodiphenyl (X) and found it to be more active than the parent amine.

However, the introduction of the fluoro-substituent also produced a profound change in the pattern of tumour incidence. With 4-aminodiphenyl itself the commonest tumours were intestinal ; but with the 4′-fluoro- derivative, tumours appeared predominantly in the liver and kidney.

Aromatic amines of some other types are also cancer producing. 2-Amino-anthracene (XI) is closely related to 2-aminofluorene, and it has produced liver tumours in mice receiving the compound by subcutaneous injection (Shear, 1938). Tumours of the skin have also been produced in rats following prolonged skin-painting with an acetone solution (Bielschowsky, 1946).

XI

XII

2-Acetylaminophenanthrene (XII) has produced tumours of the mammary gland, ear duct, and intestinal epithelium, and also showed a moderate leukemogenic activity. 3-Acetylaminophenanthrene also produced tumours of the mammary gland, ear duct and small intestine (Miller, Sandin, Miller and Rusch, 1955).

All these amines are, of course, also related to benzidine (XIII), which has been proved to be a bladder carcinogen for man, which produces bladder tumours in the dog, and which gives a variety of tumours in the rat (Spitz, Maguigan and Dobriner, 1950 ; Walpole; Williams and Roberts, 1954). 3 : 3′-Dihydroxy-benzidine (XIV), a probable metabolite of benzidine, is also carcinogenic to rats and mice (Baker, 1950, 1953).

The aminostilbenes represent yet another type of carcinogenic amine. Relatively few members of this group have been tested for carcinogenic activity, having been chiefly studied for their growth-inhibitory activity. However,

XIII

XIV

XV

4-aminostilbene (XV), 4-dimethylaminostilbene, 4-dimethylamino-2′-methylstilbene and 1-(4′-dimethylaminophenyl)-2-(1′-naphthyl)ethylene have been found to produce a wide variety of tumours in rats when administered by subcutaneous injection. In mice the compounds were found to be far less active (Haddow, Harris, Kon and Roe, 1948).

To conclude this section, it seems that although the carcinogenic aromatic amines are of rather diverse structure, the requirements for activity are becoming clearer. For example, among the derivatives and analogues of 2-acetylaminofluorene the structural requirements for activity against the liver are summarised in the following chart which is due to Miller, Sandin, Miller and Rusch (1955).

*Inactive if*
-H H-

*Weakly active if*
-CO-
-CHOH-

*Active if*
$-NH_2$
$-NHCH_3$
$-NO_2$
$-N(CH_3)_2$

Active at sites other than liver:

*Inactive if*
-S-
-SO-
-O-
-CH=CH-
$-CH_2-CH_2-$
-H H-
$-SO_2-$
-NH

*Inactive if*
-H
-Cl

This summary would appear to indicate that a fluorene nucleus is essential for strong carcinogenic activity towards the liver ; but if other structural features are *also* changed, this would not seem to be true. 4′-Fluoro-4-aminodiphenyl does produce tumours of the liver, and in this connection it is noteworthy that

7-fluoro-2-acetylaminofluorene is much more active towards the liver of both male and female rats than 2-acetylaminofluorene. It is reasonable to conclude therefore that certain structural features may profoundly affect the distribution of the compound in the animal body and hence the distribution of tumours. On the other hand, these same structural features may have little or no effect in governing whether a certain substance is actually carcinogenic or not.

It has been suggested that the greater carcinogenic activity of derivatives of fluorene *versus* diphenyl derivatives is associated with the $-CH_2-$ bridge which helps to maintain a coplanar arrangement of the benzene nuclei (Miller, Miller, Sandin and Brown, 1949 ; Sandin *et al.*, 1952). In this connection attention has been called to the striking difference in the incidence and distribution of tumours produced by 2-acetylaminofluorene, and 4-acetylaminodiphenyl, and the lack of activity in 2-methyl-4-acetylaminodiphenyl and 2′-methyl-4-acetylaminodiphenyl (Table I). Spectrographic evidence confirms the suggestion that planarity is greater in 2-acetylaminofluorene than in 4-acetylaminodiphenyl, and it also indicates that the two methyl derivatives are less planar than 4-acetylaminodiphenyl itself.

TABLE I.*—*Carcinogenic Activities of 2-Acetylaminofluorene and Certain Diphenyl Derivatives.*†

| | | | Number of rats with tumours in | | | |
|---|---|---|---|---|---|---|
| Compound. | Sex. | Number of rats studied. | Liver. | Mammary gland. | Ear duct. | Small intestine. |
| 2-Acetylaminofluorene | M | 26 | 22 | 0 | 10 | 13 |
| | F | 25 | 0 | 21 | 20 | 5 |
| 4-Acetylaminodiphenyl | M | 15 | 0 | 0 | 0 | 0 |
| | F | 15 | 0 | 11 | 2 | 0 |
| 2-Methyl-4-acetylaminodiphenyl | M | 9 | 0 | 0 | 0 | 0 |
| | F | 9 | 0 | 0 | 0 | 0 |
| 2′-Methyl-4-acetylaminodiphenyl | M | 9 | 0 | 0 | 0 | 0 |
| | F | 9 | 0 | 0 | 0 | 0 |

* Sandin *et al.* (1952).

† Final tumour incidences in rats fed 1·62 m. moles of compound per kg. of a grain diet for 8 months and the grain diet alone for an additional 2 months.

Nevertheless planarity cannot be a sole determining factor for pronounced activity, and in particular for activity against the liver, for the methyl derivative, 3 : 2′-dimethyl-4-aminodiphenyl, in which there is just as much restriction to planarity, is more active than 4-aminodiphenyl itself. Moreover, as mentioned above, the introduction of a fluoro-substituent seems to increase the activity, particularly towards the liver, and this substitution can have little effect on the planarity of the molecule.

*Metabolism of aromatic amines*

When 2-naphthylamine (XVI) is administered to dogs it is oxidised to 2-amino-1-naphthol (XVII) and excreted as ethereal sulphate (XVIII) (Wiley, 1938 ; Bonser, Clayson and Jull, 1951).

XVI

XVII

XVIII

In rats, rabbits and monkeys, however, the administration of 2-naphthylamine by subcutaneous injection was found to be followed by the excretion in the urine of 2-acetylaminonaphthalene and 2-acetylamino-6-hydroxynaphthalene, together with unchanged amine (Dobriner, Hofmann and Rhoads, 1941). Subsequently, it has been found that some 2-naphthylamine-1-sulphuric acid is also excreted by rats. It has also been found that administration of 2-acetylaminonaphthalene to rats by subcutaneous injection, is followed by the excretion of 2-acetylamino-6-hydroxynaphthalene (Manson and Young, 1950).

These metabolic transformations of 2-naphthylamine in the rat are summarised in the following chart in which conversions which have been shown to take place are indicated by solid arrows. Possible intermediary reactions are indicated by broken arrows.

It seems, therefore, that 2-naphthylamine is metabolised by two distinct routes in laboratory animals, one leading to a 2-amino-1-naphthol derivative, and the other to a 2-amino-6-naphthol derivative. Clayson (1950) has developed a method for the estimation of 2-amino-1-naphthol conjugates in the urine, and Bonser, Clayson and Jull (1951) have used the procedure to determine the urinary excretion of the amine in different species. The results are summarised in Table II.

It seems that there is an approximate correlation between the tumour-susceptibility of the species and the amount of 2-amino-1-naphthol conjugate excreted. The dog, which is particularly susceptible to 2-naphthylamine carcinogenesis excretes a high percentage of the dose as 2-amino-1-naphthol conjugates. On the other hand, the mouse, rat and rabbit, which are less susceptible, excrete smaller quantities in this form.

Bonser, Clayson and Jull concluded that 2-amino-1-naphthol is the actual carcinogen and that the parent amine, 2-naphthylamine, is without direct activity. 2-Aminno-1-naphthol hydrochloride was accordingly tested as a local carcinogen using a method involving the surgical introduction of paraffin wax pellets containing the material into the lumen of the bladder of the mouse. Under these conditions 2-amino-1-naphthol hydrochloride proved to be an active carcinogen of the same

TABLE II.—*Excretion of 2-amino-1-naphthol Conjugates in Urine of Various Species**

| Species. | Dose (mg/kg.). | Route of administration. | Recovery of metabolite (percentage of dose). |
|---|---|---|---|
| Dog | 30 | By mouth | 55–70 |
| | 120 | ,, ,, | 30–45 |
| Cat | 50 | ,, ,, | 30–50 |
| Mouse | 120 | ,, ,, | 20–40 |
| Rat | 25 | Intraperitoneal | 6–9† |
| | 150 | ,, | 12–15† |
| Rabbit | 200 | By mouth | about 5 |

* Bonser, Clayson and Jull (1951).
† The lower value was obtained in rats given a high-protein (20 per cent) diet, and the higher value in rats given a low-protein (5 per cent) diet.

order of potency as methylcholanthrene ; but 2-naphthylamine itself proved to be inactive when tested in this way. Moreover, the subcutaneous injection of 2-amino-1-naphthol hydrochloride in oil has given a few sarcomas in mice and rats (Bonser, Clayson, Jull and Pyrah, 1952). (It may be noted that the rat is not a suitable test animal for investigations involving implantation of wax pellets into the bladder. Paraffin wax itself can cause tumours in the bladder of this species (Bonser, Clayson, Jull and Pyrah, 1953).)

Largely on the basis of these results, it has been suggested that all carcinogenic amines may owe their activity to the fact that they are converted, at least in part, to *ortho*-hydroxyamines (Walpole, Williams and Roberts, 1952 ; Clayson, 1953). Certain structural features, as well as the species of animal under test, may affect the proportion of *ortho*-hydroxyamine formed, and may therefore be of prime importance. For example, it has been suggested that those aromatic amines which have a blocked *para* position will tend to hydroxylate to a greater extent in the *ortho*- position, and thus give rise to carcinogenic substances. Furthermore it may well be that 1-phenylazo-2-naphthol (XIX) and 1-*o*-tolylazo-2-naphthol (XX) are reduced *in vivo* to 1-amino-2-naphthol, and this might account for the undoubted carcinogenic activity of these food-colouring-matters in mice (Kirby and Peacock, 1949 ; Bonser, Clayson and Jull, 1954).

XIX

XX

In order to test this general hypothesis, 2-aminophenol, 3-amino-2-naphthol hydrochloride, and 1-amino-2-naphthol-4-sulphonic acid (sodium salt) were tested by the bladder-implantation technique in mice (British Empire Cancer

Campaign, 1953) but were found to be inactive. An impure specimen of 1-amino-2-naphthol hydrochloride did give tumours however, as did 1-phenylazo-2-naphthol and 4-hydroxy-3-aminodiphenyl hydrochloride.

Walpole, Williams and Roberts (1952) pointed out that the metabolic yield of *o*-hydroxyamines is increased by *o*-methylation and by N : N-dimethylation, and they therefore suggested that *o*-toluidine and dimethylaniline, but not aniline, should produce bladder tumours in the dog. However, this suggestion has been considerably weakened by the failure to confirm any local carcinogenic action with *o*-aminophenol. Moreover, Druckney and Schmähl (1954) and Druckney, Schmähl and Reiter (1954) have tested the three isomeric N : N-dimethyltoluidines in rats in doses of up to 10 g. per animal over the entire life span. No chronic toxic effects were observed, the average life span was not diminished and no carcinogenic activity was observed. Finally, 2-hydroxy-4-dimethylaminoazobenzene and 2′-hydroxy-4-dimethylaminoazobenzene, both of which would be expected to give *o*-hydroxyamines on metabolic reduction of the azo- group, have failed to give tumours (Badger, and Lewis, 1952).

Further work is clearly necessary in order to test this interesting hypothesis. In support it may be noted that although 2-amino-7-hydroxyfluorene has been identified as a metabolite of 2-acetylaminofluorene in rats (Bielschowsky, 1945*b*) ; in recent work using carrier techniques, 1-hydroxy-, 3-hydroxy-, and 5-hydroxy-2-acetylaminofluorene have also been identified as metabolites in the urine of rats fed 2-acetylaminofluorene-9–$C^{14}$ (Weisburger and Weisburger, 1954, 1955). The testing of these substances as local carcinogens will therefore be awaited with interest. Similarly 2-hydroxy-3-acetylaminofluorene has been found to be a metabolite of 3-acetylaminofluorene in the rat (Weisburger, 1955).

Furthermore, benzidine seems to be metabolised to 3-hydroxybenzidine and 4 : 4′-diamino-3-diphenylyl hydrogen sulphate in the dog (British Empire Cancer Campaign, 1954, p. 223 ; Bradshaw and Clayson, 1955). In humans it is metabolised, at least in part, to 3 : 3′-dihydroxybenzidine, and when fed to rats this metabolite rapidly produced intestinal tumours (Baker, 1950, 1953 ; Adler, 1908 ; Walpole, Williams and Roberts, 1952). On the other hand, 3 : 3′-dihydroxybenzidine proved inactive when tested by the bladder-implantation technique in in mice (British Empire Cancer Campaign, 1953) so the significance of the former result is in some doubt.

In dogs, 4-aminodiphenyl is metabolised to 4-amino-3-diphenylyl hydrogen sulphate. When the urine was hydrolysed by boiling with acid, 3-hydroxy-4-aminodiphenyl was identified (Bradshaw and Clayson, 1955). All these reports therefore support the view that *ortho*-hydroxylation is of considerable importance in the carcinogenic process.

### CHOLESTEROL AND RELATED COMPOUNDS

Although the discovery of 3 : 4-benzopyrene and of other similar carcinogens has done much to explain the incidence of some occupational and environmental cancers, the great majority of human cancers remain " spontaneous " and unexplained. In this connection the hypothesis that a chemical carcinogen may be formed *in vivo* by some abnormal mechanism should clearly be investigated, and work has proceeded along several different lines.

In 1932 Kennaway and Cook suggested that a polycyclic aromatic hydrocarbon might arise from certain steroids by some abnormal mechanism. Soon afterwards, 20-methylcholanthrene was prepared from deoxycholic acid, albeit by laboratory methods (Cook, 1933 ; Cook and Haslewood, 1933, 1934, 1935 ; Wieland and Dane, 1933), and this 1 : 2-benzanthracene derivative was soon found to be a very potent carcinogen (Barry *et al.*, 1935).

Methylcholanthrene has also been prepared from cholic acid and from cholesterol ; but there is no evidence that transformations of this type do in fact proceed *in vivo*. At the moment, this work must be considered solely as an interesting speculation. There is, however, some evidence that deoxycholic acid itself is carcinogenic when given to mice by injection in sesame oil (Badger, Cook, Hewett, Kennaway, Kennaway and Martin, 1942).

Another suggestion has been that polycyclic aromatic hydrocarbons might arise from steroids, etc., during the cooking of food, and this hypothesis has also been examined (Peacock, 1947). A carcinogenic tar has certainly been produced by the pyrolysis of cholesterol (Kennaway and Sampson, 1928), but the temperature involved was much greater than could occur in cooking. More recently, Falk, Goldfein and Steiner (1949) have studied the pyrolysis of cholesterol at 360°, and the following compounds were identified : cholestenone, dicholesteryl ether, 3 : 5-cholestadiene, the naphthalene derivative of cholesterol, the phenanthrene derivative of cholesterol, methylcyclopentenophenanthrene and chrysene. Except for cholestenone, all these products may be considered as intermediates in the transformation of cholesterol to methylcholanthrene ; but the latter compound was not found.

An investigation of a different kind was initiated by Hieger (1946, 1947, 1949) who has shown that cholesterol (XXI) has definite if weak carcinogenic activity. The first experiments were carried out using a commercial cholesterol, which gave 25 sarcomas in 436 mice. Subsequently, however, it was found that purified cholesterol is also cancer-producing (Hieger and Orr, 1954).

Fieser (1954) has pointed out that as the total quantity of cholesterol in a man weighing 65 kg. is approximately 210 g., or 0·3 per cent of the wet weight, it is unlikely that it can have the properties of a carcinogen. He suggested that the observed activity must be due to a transformation product. In this connection it is noteworthy that a crude progesterone preparation, prepared by permanganate oxidation of cholesterol dibromide, and debromination, produced tumours in 32 per cent of the mice tested (Bischoff and Rupp, 1946 ; Spielman and Meyer, 1939).

Reasoning in terms of the chemical reactivity required for carcinogenic activity, Fieser (1954) suggested that $\Delta^5$-cholestene-3-one (XXII) might be the true carcinogen formed from cholesterol. It has recently been found (Fieser, Greene, Bischoff, Lopez and Rupp, 1955) that the hydroperoxide (XXIII) derived from this compound is an active carcinogen. This hydroperoxide, 6 $\beta$-hydroperoxy-$\Delta^4$-cholestene-3-one (XXIII), was given by subcutaneous injection to 32 mice, and at the age of 12 months fibrosarcomas had appeared at the site of injection in 13 of the mice treated (average tumour age, 9·6 months) and 17 of the remaining mice were still alive. Fieser's views on the chemical reactivity requirements for carcinogenic activity have therefore been supported in a very satisfactory way, and further work in this field will be awaited with interest. It may be added that there is some indirect evidence that $\Delta^5$-cholestene-3-one can be formed from cholesterol in the body.

XXI

XXII

XXIII

ALKYLATING AGENTS

*Nitrogen and sulphur mustards*

Various war-time studies showed that the nitrogen and sulphur mustards resemble ionizing radiations in many of their biological effects, particularly in regard to the inhibition of cell division and growth, the production of mutations and chromosome damage. Compounds of this nature have accordingly been extensively investigated as it was hoped that an effective tumour-inhibitor might be found. A few have also been tested for carcinogenic activity.

The nitrogen mustards do not seem to be carcinogenic to the skin of mice (Fell and Allsopp, 1948; Narpozzi, 1953); but they produce tumours rather slowly when administered by injection (Boyland and Horning, 1949; Griffin, Brandt and Tatum, 1950, 1951).

XXIV

XXV

XXVI

Boyland and Horning used two groups of twenty stock mice. One group was given an aqueous solution of methyl di(2-choroethyl)-amine hydrochloride (XXIV) by subcutaneous injection and received 1·0 mg./kg. at weekly intervals for 50 weeks. The second group received a similar injection of tri(2-chloroethyl)amine hydrochloride (XXV) ; but the injections were stopped after 10 weeks as only 4 mice remained alive. Of the 14 mice from the two groups which survived more than 250 days, 10 had tumours. These included lung tumours (8), lymphosarcomas (2), a uterine fibromyoma and a spindle-celled sarcoma at the site of injection.

In strain A mice, Heston (1949) obtained tumours after 16 weeks in 100 per cent of the animals receiving a total dose of 0·1 mg. of methyl di(2-chloroethyl) amine hydrochloride. Sulphur mustard (XXVI) has also been shown to give lung tumours in a high percentage of the mice receiving the material by subcutaneous injection (Heston, 1950, 1953), and it is also carcinogenic in the rat (Haddow, 1953).

Few aromatic mustards seem to have been tested, but Haddow (1953) has reported that pronounced carcinogenic activity (mainly after subcutaneous injection, but also following feeding) has been shown by several compounds (XXVII–XXXI) of this type.

XXVII

XXVIII

XXIX

XXX

XXXI

The mustards are rather reactive substances, especially in polar solvents. They react with water, with anions and with bases, and in general they can be described as powerful alkylating agents for functional groups such as –OH, $-NH_2$, etc. As such functional groups occur in nucleic acids, in enzymes, and in other important constituents of the cell, the biological activity of the mustards may be intimately related with their chemical reactivity towards such centres. In this connection it may be noted that Ross (1949, 1953) found that there is a good correlation between the rates of hydrolysis of a series of di(2-chloroethyl)amines, $R.N(CH_2CH_2Cl)_2$, and their activity as tumour inhibitors, the most reactive substances being the most active tumour-inhibitors.

The inductive effect of the hetero-atom would be expected to facilitate the removal of the halogen atom to give the carbonium ion (XXXIII), and considerable evidence has accumulated to indicate that this ion is, in fact, the reacting species (Ross, 1953 ; Alexander, 1954). With aliphatic (but not with aromatic) derivatives, this carbonium ion may be stabilised by passing into the cyclic ethyleneimonium ion (XXXIV).

XXXII XXXIII XXXIV

Proof that ionisation does occur was provided by Seligman, Rutenberg and Friedman (1949) who administered diethyl (2-iodoethyl)amine-$I^{131}$. The resulting radioactivity pattern was found to be almost identical with that shown following administration of radioactive sodium iodide.

This picture of the nitrogen and sulphur mustards as biological alkylating agents suggested that other alkylating agents might also possess similar biological actions. An hypothesis (Goldacre, Loveless and Ross, 1949) that the action might be associated with a cross-linkage reaction directed attention to known cross-linking agents for textile fibres ; but subsequent work has shown that some mono-functional alkylating agents have carcinogenic activity.

In these groups of alkylating agents the following structural features appear to be the significant ones :

$-S\,.\,CH_2CH_2Cl$ . . . sulphur mustard

$>N\,.\,CH_2CH_2Cl$ . . . nitrogen mustards

 . . . epoxides

 . . . ethylene imines

$-CH_2\,.\,O\,.\,SO_2CH_3$ . . . methane sulphonates.

*Ethyleneimine derivatives*

Several N-substituted ethyleneimine derivatives have been tested for carcinogenic activity in both mice and rats (Walpole, Roberts, Rose, Hendry and Homer, 1954), and the results are summarised in Table III.

The compounds were generally tested by subcutaneous injection of their solutions in arachis oil, but the doses and frequencies of injection were not standardised, and it is therefore difficult to assign accurate relative potencies. It seems however that ethyleneimine (XXXV) itself is carcinogenic and that a number of acylethyleneimines (XXXVI) are likewise active. The most potent compounds produced tumours in a high percentage of the rats tested after a latent period of about five months.

XXXV

XXXVI

XXXVII

TABLE III.—*Carcinogenic Activity of Ethyleneimine Derivatives.**

| Compound. | Carcinogenic activity.† Mice. | Carcinogenic activity.† Rats. |
|---|---|---|
| Ethyleneimine $HN\langle\begin{smallmatrix}CH_2\\ \vert\\ CH_2\end{smallmatrix}$ | .. | ++ |
| Acetylethyleneimine $CH_3CO\,.\,N\langle\begin{smallmatrix}CH_2\\ \vert\\ CH_2\end{smallmatrix}$ | + | ++ |
| Butyrylethyleneimine $CH_3(CH_2)_2\,.\,CO\,.\,N\langle\begin{smallmatrix}CH_2\\ \vert\\ CH_2\end{smallmatrix}$ | ± | ++ |
| Caproylethyleneimine $CH_3(CH_2)_4\,.\,CO\,.\,N\langle\begin{smallmatrix}CH_2\\ \vert\\ CH_2\end{smallmatrix}$ | + | ++ |
| Nonanoylethyleneimine $CH_3(CH_2)_7\,.\,CO\,.\,N\langle\begin{smallmatrix}CH_2\\ \vert\\ CH_2\end{smallmatrix}$ | + | ++ |
| Lauroylethyleneimine $CH_3(CH_2)_{10}\,.\,CO\,.\,N\langle\begin{smallmatrix}CH_2\\ \vert\\ CH_2\end{smallmatrix}$ | .. | ± |
| Myristoylethyleneimine $CH_3(CH_2)_{12}\,.\,CO\,.\,N\langle\begin{smallmatrix}CH_2\\ \vert\\ CH_2\end{smallmatrix}$ | ++ | ++ |
| Stearoylethyleneimine $CH_3(CH_2)_{16}\,.\,CO\,.\,N\langle\begin{smallmatrix}CH_2\\ \vert\\ CH_2\end{smallmatrix}$ | .. | ++ |
| $CH_3(CH_2)_7\,.\,CH = CH\ (CH_2)_7\,.\,CO\,.\,N\langle\begin{smallmatrix}CH_2\\ \vert\\ CH_2\end{smallmatrix}$ | .. | +++ |
| N : N-Dimethylstearamide $CH_3(CH_2)_{16}\,.\,CO\,.\,N\langle\begin{smallmatrix}CH_3\\ \vert\\ CH_3\end{smallmatrix}$ | .. | + |

* Walpole, Roberts, Rose, Hendry and Homer (1954).
† Dissolved in arachis oil and injected subcutaneously.

Some attempt was also made to test the analogous ethyleneiminosulphonyl alkanes (XXXVII), but the three compounds tested ($R = C_7H_{15}$, $C_5H_{11}$ and $C_3H_7$) all proved to be inactive.

XXXVIII

XXXIX

A few ethyleneimine derivatives of a different type hae also been tested. For example, the heterocyclic compound (XXXVIII) was found to be a very potent carcinogen when tested by injection into rats (Walpole, Roberts, Rose, Hendry and Homer, 1954). Triethylenemelamine (*tris*ethyleneimino-*s*-triazine, XXXIX) has been found to induce multiple pulmonary tumours in Strain A mice (Shimkin, 1954), but it seems to be inactive in stock mice (Hendry, Homer, Rose and Walpole, 1951). On the other hand it gives sarcomas at the site of injection in rats and, indeed, is very potent in this respect (Walpole, Roberts, Rose, Hendry and Homer, 1954). Moreover, Roe and Salaman (1955) have found that it has an initiating action on the skin of mice, tumours being produced by subsequent treatment with a promoting agent (croton oil).

It has been suggested (Walpole, Roberts, Rose, Hendry and Homer, 1954) that ethyleneimine may be regarded as the ultimate carcinogen, the function of the acyl group being merely to modulate chemical reactivity and to provide an electrically neutral derivative capable of diffusing readily into the cell. The compounds may be regarded as alkylating agents and the following general scheme may serve as a basis for further work :

$$R\ N\begin{array}{l}\diagup CH_2\\ \quad\ |\\ \diagdown CH_2\end{array} + \text{Cell} - H \rightarrow \text{Cell} - CH_2CH_2NHR$$

On the other hand, the same authors have found that the three-membered ring system is not essential for carcinogenic activity as N, N-dimethylstearamide (XL) has some carcinogenic activity. However, it is less active than the corresponding cyclic compound, stearoylethyleneimine.

$$CH_3(CH_2)_{16}\ .\ CO\ .\ N\begin{array}{l}\diagup CH_3\\ \diagdown CH_3\end{array}$$

XL

*Trimethylolmelamine*

Polymethylolamides have been used to modify the mechanical properties of textile fibres, and a number of such substances were therefore submitted for test as tumour-inhibiting agents.

XLI

Trimethylolmelamine (XLI) has been tested as a carcinogen in mice and rats (Hendry, Rose and Walpole, 1951). Subcutaneous injection into stock mice resulted, after nine months, in one lymphoid leukaemia, no other mouse showing any sign of malignant disease. Of the 30 rats injected, one developed a highly malignant adenocarcinoma, one a spindle-celled sarcoma at the site of injection, and one developed a lymphoid leukaemia. The authors point out that these tumours may have been spontaneous and do not regard the above results as unequivocal evidence for carcinogenic activity.

*Bis-Epoxides*

Following the cross-linking hypothesis of Goldacre, Loveless and Ross (1949) bis-epoxides were found to possess the tumour-inhibiting activity and to produce the chromosome abnormalities characteristic of the mustards. Only two compounds of this type seem to have been tested for carcinogenic activity.

XLII

XLIII

Vinyl *cyclo*hexane dioxide (XLII) has been reported to be a carcinogen for both mice and rats (Hendry, Homer, Rose and Walpole, 1951). This substance was administered to rats by intraperitoneal injection of its solution in arachis oil and a mixed cell sarcoma was obtained in one animal at seven months. When tested in mice by application to the skin, this dioxide seemed to be highly carcinogenic. Of the 20 mice receiving the compound, nine developed malignant tumours and two developed papillomata which regressed when the treatment was stopped. However, Walpole, Roberts, Rose, Hendry and Homer (1954) have repeated the experiment with a more highly purified sample of the diepoxide and have failed to obtain tumours. In the circumstances the carcinogenicity of vinyl *cyclo*hexane dioxide must remain in doubt.

Butadiene dioxide (XLIII) gave no tumours in mice (Hendry, Homer, Rose and Walpole, 1951). A few tumours were observed in rats following intraperitoneal injection of its solution in arachis oil. Here again, however, further experiments are necessary to confirm the carcinogenic activity of this substance.

*Propiolactone*

The simple aliphatic compound propiolactone (XLIV) was found to be mutagenic (Smith and Srb, 1951) and was therefore tested as a carcinogen. It gave a high yield of sarcomata following subcutaneous injection in rats and must be rated as a moderately potent carcinogen (Walpole, Roberts, Rose, Hendry and Homer, 1954).

XLIV

Roe and Salaman (1955) found that it is not carcinogenic to the skin of the mouse; but they were able to show that it does have an initiating action. That is, treatment with propiolactone, followed by croton oil, did produce tumours.

*Methane Sulphonates*

No complete report has appeared on the carcinogenic activity of compounds of this type. It appears, however, that compounds of general formula (XLV) and (XLVI) are active. 1 : 4-Dimethanesulphonoxybutane is reported to be outstanding in this respect (Haddow, 1953). Shimkin (1954) has found that it does not increase the incidence of pulmonary tumours in strain A mice.

XLV

$CH_3SO_2O\ .\ (CH_2)_n\ .\ O\ SO_2CH_3$

XLVI

### OTHER ORGANIC CARCINOGENS

*Senecio Alkaloids*

Plants of the *Senecio* species are widely used by South African negroes for medicinal purposes, and it seemed probable that this practice might have some bearing on the high incidence of primary liver cancer among these people. In order to test this hypothesis, Cook, Duffy and Schoental (1950) administered the crude alkaloids from *Senecio jacobaea* L. (the common ragwort) in the drinking water, to albino rats. Liver tumours were observed in 3 rats which survived more than eight months of the treatment.

*Senecio jacobaea* is not itself a native of South Africa, but the alkaloidal constituents are similar to those found in the local species which are commonly used by the natives. More recently, the pure alkaloids retrorsine and isatidine, which are known to occur in South African *Senecio* plants, have also been shown to produce liver tumours (Schoental, Head and Peacock, 1954). In these experiments 58 rats survived the treatment with retrorsine, isatidine or the mixed alkaloids of *Senecio jacobaea* for more than 10 months. Of these, 45 showed changes in the liver ranging from hyperplasia to neoplasia. It is of some interest that tumours were produced in animals receiving a fully adequate diet. A deficient diet of the type known to favour tumour formation by the use of azo-dyes did not prove favourable with the *Senecio* alkaloids.

The alkaloid seneciphylline, also isolated from *Senecio jacobaea*, has been shown to produce liver tumours in fowls (British Empire Cancer Campaign, 1954, p. 4).

The chemistry of the senecio alkaloids (the structures of most being known only imperfectly) has been reviewed by Leonard (1950).

*Tannic acid*

When injected subcutaneously in rats, tannic acid produces cirrhosis, hepatomas and cholangiomas in the liver ; but no local carcinogenic action could be demonstrated (Korpássy and Kovacs, 1949 ; Korpássy and Mosonyi, 1950). The carcinogenic action on the liver was found to be retarded by a high-casein-low-fat diet, and accelereated by a low-casein high-fat diet (Korpássy and Mosonyi, 1953).

*Chloroform and carbon tetrachloride*

The repeated oral administration of carbon tetrachloride in olive oil to mice of various strains has been shown to produce hepatomas in a very high percentage of the animals treated (Edwards, 1941 ; Edwards and Dalton, 1942 ; Edwards, Edwards, Heston and Dalton, 1942 ; Eschenbrenner and Miller, 1944).

Similarly the repeated oral administration of chloroform also produces hepatomas and cirrhosis of the liver, provided the individual doses are large enough to produce liver necrosis (Eschenbrenner and Miller, 1945).

These findings are of some importance in view of the widespread medical and industrial use of these materials. It is noteworthy that some hepatic injury has been reported in workers employed in the manufacture of carbon tetrachloride insecticide and fire extinguisher bombs (Sassi and Paruccini, 1954).

The mode of action of these agents remains obscure but it seems that the carcinogenic activity is completely unrelated to the narcotic activity.

*Urethanes*

The intraperitoneal injection of ethyl urethane (ethyl carbamate, XLVII) has been markedly to increase the incidence of lung tumours in mice (Nettleship, Henshaw and Meyer, 1943).

$$H_2N\ .\ COOC_2H_5$$

XLVII

The effect has been found in all the strains of mice tested, but some strains were found to be more susceptible than others. It seems that this susceptibility parallels the susceptibility of the strain to spontaneous pulmonary tumours (Larsen, 1946 ; Orr, 1947 ; Cowen, 1947). The effect is unrelated to the anaesthetic action of urethane, and a variety of other hypnotics proved ineffective in increasing the incidence of lung tumours. On the other hand a single anaesthetic dose of urethane sometimes proved effective ; moreover the induction period may be as short as two months (Henshaw and Meyer, 1944).

Ethyl urethane has also been found to have a carcinogenic action in rats, whether administered orally or by intraperitoneal injection (Jaffé, 1947 ; Guyer and Claus, 1947). In both cases a high incidence of lung tumours was observed.

Other esters of carbamic acid have also been tested as pulmonary carcinogens, but the ethyl ester seems to be the most potent. The relative potencies of the ethyl, *iso*propyl and *n*-propyl esters, for example, were found to be of the order of 81, 4 and 1 respectively. Trichloroethyl carbamate was found to be slightly carcinogenic (and toxic); but the methyl-monochloroethyl-, *n*-butyl- and *iso*amyl esters were found to be inactive at the dosage levels employed (Larsen, 1947).

The effect of alkyl substitution on the nitrogen atom has also been studied (Larsen, 1948), and in general, changes of this type were found to decrease the activity as pulmonary carcinogens. Ethyl N-methyl carbamate was found to be much less effective than ethyl carbamate itself; and N,N-dialkylated derivatives were slightly less effective than the mono-alkyl compounds. With the exception of the *N*-isopropyl compound, activity decreased as the length of the alkyl group was increased.

In addition to this carcinogenic action towards the lung, it has recently been shown that urethane produces a pre-neoplastic change when applied to the skin of mice. When applied alone to the skin it produces no local tumours; but when such application is followed by treatment with a promoting agent (croton oil) tumours are produced (Salaman and Roe, 1953; Roe and Salaman, 1954). This tumour-initiating action of urethane can be inhibited by the simultaneous administration of glycine and formate, two purine precursors (Roe, 1955). This observation lends some support to the hypothesis that urethane acts by competing with one or more of the precursors in purine synthesis, perhaps leading to an unphysiological purine-like substance (Roe, 1955).

*Dulcin*

The synthetic sweetening agent Dulcin (*p*-phenetylurea, XLVIII) has some structural similarities to the urethanes, but this is largely superficial.

XLVIII

Fitzhugh and Nelson (1950) have found that this substance produces liver tumours in albino rats fed at dosage levels of 0·1 per cent and above. At 0·5 and 1 per cent, Dulcin retarded the growth rate, increased the mortality rate and produced dark red spleens.

*Polymers*

In 1941 Turner found that sarcomas are produced in rats following the subcutaneous implantation of Bakelite disks. Of 9 rats which survived longer than 20 months, 4 developed sarcomas at the site of the disk. It has since been found that several other natural and synthetic polymers can produce similar tumours: these include cellophane, polyethylene, polyvinylchloride, polystyrene, polymethyl methacrylate, polytetrafluoroethylene and ε-polycaprolactam (Oppenheimer, Oppenheimer and Stout, 1948, 1952, 1954; Druckrey and Schmähl, 1954; Laskin, Robinson, and Weinmann, 1954).

The pure polymers seem to be as effective as the commerical products, and benzene extracts of such polymers have produced no tumours. Implanted quartz sand usually produced tumours; but glass was found to be ineffective.

The carcinogenic activity of these polymers is somewhat surprising in view of the stability of these materials. Nevertheless polymers do degrade slowly, and it is not unreasonable to suppose that the resulting free radicals will react with important cellular constituents (Oppenheimer, Oppenheimer, Danishefsky, Stout and Eirich, 1955). The polymers do not seem to be very potent carcinogens, producing tumours only after a long latent period. Nevertheless the observations are of considerable importance in view of the use of various plastics in surgery.

### SUMMARY

1. Chemical carcinogens other than the polycyclic aromatic hydrocarbons and the azo-compounds, have been reviewed.

2. Inorganic carcinogens include derivatives of arsenic, beryllium, chromium, cobalt, nickel and zinc.

3. Many aromatic amines are cancer-producing. It seems that small changes in chemical structure may affect the distribution of the amine in the body and thus alter the distribution of tumours produced. Hormonal influences also have a profound effect on the distribution.

4. *Ortho*-hydroxylation of aromatic amines is of considerable importance in the carcinogenic process ; but not all *o*-hydroxyamines are cancer-producing.

5. Among the cholesterol derivatives, the recent discovery that 6β-hydroperoxy-$\Delta^4$-cholestene-3-one is a carcinogen is of considerable importance.

6. Nitrogen and sulphur mustards, ethyleneimine derivatives, methane sulphonates and propiolactone appear to be carcinogenic ; but the present evidence is inconclusive in regard to trimethylolamine and the bis-epoxides.

7. Several *Senecio* alkaloids are active in producing cancer of the liver, and tumours in the same organ are produced by tannic acid, chloroform, carbon tetrachloride, and by the synthetic sweetening agent Dulcin.

8. Certain urethanes (especially ethyl urethane) produce lung tumours in mice. Ethyl urethane has also been shown to have an initiating action on the skin of mice.

9. Several natural and synthetic polymers produce sarcomas following subcutaneous implantation.

### REFERENCES

Adler, O.—(1908) *Arch. exp. Path. Pharmak.*, **58**, 167.

Alexander, P.—(1954) " Advances in Cancer Research ", **2**, 1. New York (Academi Press).

Anissimova, V.(1939) *Amer. J. Cancer*, **36**, 229.

Arhelger, S. W. and Kremen, A. J.—(1951) *Surgery*, **30**, 977.

Armstrong, E. C. and Bonser, G. M.—(1944) *J. Path. Bact.*, **56**, 507.—(1947) *Ibid.*, **59**, 19.

Badger, G. M.—(1948) *Brit. J. Cancer*, **2**, 309.

*Idem*, Cook, J. W., Hewett, C. L., Kennaway, E. L., Kennaway, N. M. and Martin, R. H.—(1942) *Proc. Roy. Soc.*, B, **131**, 170.

*Idem* and Lewis, G. E.—(1952) *Brit. J. Cancer*, **6**, 270.

*Idem*, Lewis, G. E., and Reid, R. T. W.—(1954) *Nature*, **173**, 313.

Baetjer, A. M.—(1950) *Arch. industr. Hyg.*, **2**, 487, 505.

Bagg, H. J.—(1936) *Amer. J. Cancer*, **26**, 69.

Baker, K.—(1950) *Acta Un. int. Cancer*, **1**, 46. (1953) *Cancer Res.*, **2**, 137.

BARNES, J. M.—(1950) *Lancet*, i, 463.
*Idem*, DENZ, F. A. AND SISSONS, H. A.—(1950) *Brit. J. Cancer*, **4**, 212.
BARRY, G., COOK, J. W., HASLEWOOD, G. A. D., HEWETT, C. L., HIEGER, I. AND KENNAWAY, E. L.—(1935) *Proc. Roy. Soc.*, B. **117**, 318.
BARSOTTI, M. AND VIGLIANI, E. C.—(1952) *Arch. industr. Hyg.*, **5**, 234.
BERENBLUM, I. AND BONSER, G. M.—(1937) *J. industr. Hyg.*, **19**, 86.
BIDSTRUP, P. L.—(1950) *Arch. belges Méd. soc.*, **8**, 500.—(1951) *Brit. J. industr. Med.*, **8**, 302.
BIELSCHOWSKY, F.—(1944*a*) *Brit. J. exp. Path.*, **25**, 1.—(1944*b*) *Ibid.*, **25**, 90.—(1945*a*) *Ibid.*, **26**, 270.—(1945*b*) *Biochem. J.*, **39**, 287.—(1946) *Brit. J. exp. Path.*, **27**, 54.—(1947) *Brit. med. Bull.*, **4**, 382.—(1949) *Brit. J. Cancer*, **3**, 547.
*Idem* AND HALL, W. H.—(1953) *Brit. J. Cancer*, **7**, 358.
BISCHOFF, F. AND RUPP, J. J.—(1946) *Cancer Res.*, **6**, 403.
BONSER, G. M.—(1943) *J. Path. Bact.*, **55**, 1.
*Idem*, CLAYSON, D. B. AND JULL, J. W.—(1951) *Lancet*, ii, 286.—(1954) *Nature*, **174**, 879.
*Iidem* AND PYRAH, L. N.—(1952) *Brit. J. Cancer*, **6**, 412.—(1953) *Ibid.*, **7**, 456.
BOURNE, H. G. AND YEE, H. T.—(1950) *Industr. Med.*, **19**, 563.
BOYLAND, E. AND HORNING, E. S.—(1949) *Brit. J. Cancer*, **3**, 118.
BRADSHAW, L. AND CLAYSON, D. B.—(1955) *Nature*, **176**, 974.
British Empire Cancer Campaign (1953) *Ann. Rep.*, **31**, 193. (1954)—*Ibid.*, **32**, 223, 4.
CANTAROW, A., STASNEY, J. AND PASCHKIS, K. E.—(1948) *Cancer Res.*, **8**, 412.
CASE, R. A. M., HOSKER, M. E., McDONALD, D. B. AND PEARSON, J. T.—(1954) *Brit. J. industr. Med.*, **11**, 75.
CASE, R. A. M. AND PEARSON, J. T.—(1954) *Ibid.*, **11**, 213.
CLAYSON, D. B.—(1950) *Biochem. J.*, **47**, xlvi.—(1953) *Brit. J. Cancer*, **7**, 460.
COOK, J. W.—(1933) *Proc. Roy. Soc.*, B **113**, 277.
*Idem*, DUFFY, E. AND SCHOENTAL, R.—(1950) *Brit. J. Cancer*, **4**, 405.
*Idem* AND HASLEWOOD, G. A. D.—(1933) *Chem. & Ind.* (*Rev.*), **52**, 758.—(1934) *J, chem. Soc.*, p. 428.—(1935) *J. Amer. chem. Soc.*, **57**, 1380.
COWEN, P. N.—(1947) *Brit. J. Cancer*, **1**, 401.
DOBRINER, K., HOFMANN, K. AND RHOADS, C. P.—(1941) *Science*, **93**, 600.
DRUCKNEY, H. AND SCHMÄHL, D.—(1954) *Acta Un. int. Cancr.*, **10**, 119 ; *Naturwissenschaffen*, **41**, 534.
*Iidem* AND REITER, A.—(1954) *Arzneimittel-Forsch.*, **4**, 365.
DUNNING, W. F., CURTIS, M. R. AND MADSEN, M. E.—(1947) *Cancer Res.*, **7**, 134.
DUPTA, D. N.—(1955) *Nature*, **175**, 257.
DUTRA, F. R. AND LARGENT, E. J.—(1950) *Amer. J. Path.*, **26**, 197.
*Iidem* AND ROTH, J. L.—(1951) *Arch. Path.*, **51**, 473.
EDWARDS, J. E.—(1941) *J. nat. Cancer Inst.*, **2**, 197.
*Idem* AND DALTON, A. J.—(1942) *Ibid.*, **3**, 19.
*Idem*, HESTON, W. E. AND DALTON, A. J.—(1942) *Ibid.*, **3**, 297.
ESCHENBRENNER, A. B. AND MILLER, E.—(1944) *Ibid.*, **4**, 385.—(1945) *Ibid.*, **5**, 251.
FALIN, L. I. AND GROMZEWA, K. E.—(1939) *Amer. J. Cancer*, **36**, 233.
FALK, H. L., GOLDFEIN, S. AND STEINER, P. E.—(1939) *Cancer Res.*, **9**, 438.
FELL, H. B. AND ALLSOPP, C. B.—(1948) *Cancer Res.*, **8**, 177.
FIESER, L. F.—(1954) *Science*, **119**, 719.
*Idem*, GREENE, T. W., BISCHOFF, F., LOPEZ, G. AND RUPP, J. J.—(1955) *J. Amer. chem. Soc.*, **77**, 3928.
FITZHUGH, O. G. AND NELSON, A. A.—(1948) *Science*, **108**, 626.—(1950) *Feder. Proc.*, **9**, 272.
FOULDS, L.—(1947) *Brit. J. Cancer*, **1**, 172.
GARDNER, L. U. AND HESLINGTON, H. F.—(1946) *Feder. Proc.*, **5**, 221.
GOLDBLATT, M. W.—(1949) *Brit. J. industr. Med.*, **6**, 65.

GOLDACRE, R. J., LOVELESS, A. AND ROSS, W. C. J.—(1949) *Nature*, **163**, 667.

GRAHAM, J. D. P. AND HOOD, J.—(1948) *Brit. J. Pharmacol.*, **3**, 84.

GRIER, R. S., NASH, P. AND FREIMAN, D. G.—(1948) *J. industr. Hyg.*, **30**, 228.

GRIFFIN, A. C., BRANDT, E. L. AND TATUM, E. L.—(1950) *J. Amer. med. Ass.*, **144**. 571.—(1951) *Cancer Res.*, **11**, 253.

GUYER, M. F. AND CLAUS, P. E.—(1947) *Cancer Res.*, **7**, 342.

HADDOW, A.—(1953) 'The Physiopathology of Cancer' (edited Homburger and Fishman) New York (Hoeber).

*Idem*, HARRIS, R. J. C., KON, G. A. R. AND ROE, E. M. F.—(1948) *Phil. Trans.*, **241**, 147.

HALL, W. H.—(1948) *Brit. J. Cancer*, **2**, 273.

HARRIS, P. N.—(1947) *Cancer Res.*, **7**, 88.

HEATH, J. C.—(1954) *Nature*, **173**, 822.

HENDRY, J. A., HOMER, R. F., ROSE, F. L. AND WALPOLE, A. L.—(1951) *Brit. J. Pharmacol.*, **6**, 357.

HENDRY, J. A., MATTHEWS, J. J., WALPOLE, A. L. AND WILLIAMS, M. H. C.—(1955) *Nature*, **175**, 1131.

HENDRY, J. A., ROSE, F. L. AND WALPOLE, A. L.—(1951) *Brit. J. Pharmacol.*, **6**, 20.

HENSHAW, P. S. AND MEYER, H. L.—(1944) *J. nat. Cancer Inst.*, **4**, 523.

HESTON, W. E.—(1949) *Ibid.*, **10**, 125.—(1950) *Ibid.*, **11**, 415.—(1953) *Proc. Soc. exp. Biol., N.Y.*, **82**, 457.

HIEGER, I.—(1946) *Cancer Res.*, **6**, 657.—(1947) *Nature*, **160**, 270.—(1949) *Brit. J. Cancer*, **3**, 123.

*Idem* AND ORR, J. W.—(1954) *Brit. J. Cancer*, **8**, 274.

HOAGLAND, M. B., GRIER, R. S. AND HOOD, M. B.—(1950) *Cancer Res.*, **10**, 629.

HUEPER, W. C.—(1938) *Arch. Path.*, **25**, 856.—(1942) " Occupational Tumors and Allied Diseases', Springfield, Illinois (Charles C. Thomas) 896 pp.—(1952) *Tex. Rep. Biol. Med.*, **10**, 167.—(1954) *Arch. Path.*, **58**, 360, 475, 645.

*Idem*, WILEY, F. H. AND WOLFE, H. D.—(1938) *J. industr. Hyg.*, **20**, 46.

*Idem*, ZUEFLE, J. H., LINK, A. M. AND JOHNSON, M. G.—(1952) *J. nat. Cancer Inst.*, **13**, 291.

JAFFÉ, W. G.—(1947) *Cancer Res.*, **7**, 107.

KENNAWAY, E. L. AND COOK, J. W.—(1932) *Chem. & Ind.* (*Rev.*), **51**, 521.

KENNAWAY, E. L. AND SAMPSON, B.—(1928) *J. Path. Bact.*, **31**, 609.

KIRBY, A. H. M.—(1947) *Brit. J. Cancer*, **1**, 68.—(1948) *Ibid.*, **2**, 294.

*Idem* AND PEACOCK, P. R.—(1949) *Glasg. med. J.*, **30**, 364.

KORPÁSSY, B. AND KOVÁCS, K.—(1949) *Brit. J. exp. Path.*, **30**, 266.

*Idem* AND MOSONYI, M.—(1950) *Brit. J. Cancer*, **4**, 411.—(1953) *Kiserl. Orvostud*, **5**, 258.

LARIONOV, L. F.—(1930) *Voprosy Onkologii, Kharkov*, **3**, 114 ; *Amer. J. Cancer*, **15**, 2832 (1931).

LARSEN, C. D.—(1946) *Cancer Res.*, **6**, 500 Proc.—(1947) *J. nat. Cancer Inst.*, **8**, 99.—(1948) *Ibid.*, **9**, 35.

LASKIN, D. M., ROBINSON, I. B. AND WEINMANN, J. P.—(1954) *Proc. Soc. exp. Biol. N.Y.*, **87**, 329.

LEONARD, N. J.—(1950) 'The Alkaloids', edited Manske and Holmes. New York (Academic Press). Vol. 1, p. 107.

MACHLE, W. AND GREGORIUS, F.—(1948) *Publ. Hlth Rep. Wash.*, **63**, 1114.

MANCUSO, T. F. AND HUEPER, W. C.—(1931) *Industr. Med.*, **20**, 358.

MANSON, L. A. AND YOUNG, L.—(1950) *Biochem. J.*, **47**, 170.

MICHALOWSKY, I.—(1928) *Virchows Arch.*, **267**, 27.

MILLER, E. C., MILLER, J. A., SANDIN, R. B. AND BROWN, R. K.—(1949) *Cancer Res.*, **9**, 504.

MILLER, J. A., SANDIN, R. B., MILLER, E. C. AND RUSCH, H. P.—(1955) *Ibid.*, **15**, 188.

MORRIS, H. P., WEISBURGER, J. H. AND WEISBURGER, E. K.—(1950) *Ibid.*, **10**, 620.

Narat, J. K.—(1925) *J. Cancer Res.*, **9**, 135.
Narpozzi, A.—(1953) *Riv. Anat. pat. Oncol. (Padova)*, **6**, 1155.
Nash, P.—(1950) *Lancet*, i, 519.
Nettleship, A., Henshaw, P. S. and Meyer, H. L.—(1943) *J. nat. Cancer Inst.*, **4**, 309.
Neubauer, O.—(1947) *Brit. J. Cancer*, **1**, 192.
Oppenheimer, B. S., Oppenheimer, E. T. and Stout, A. P.—(1948) *Proc. Soc. exp. Biol. N.Y.*, **67**, 33.—(1952) *Ibid.*, **79**, 366.—(1954) *Surg. Forum*, **4**, 672.
Oppenheimer, B. S., Oppenheimer, E. T., Danishefsky, I., Stout, A. P. and Eirich F. R.—(1955) *Cancer Res.*, **15**, 333.
Orr, J. W.—(1947) *Brit. J. Cancer*, **1**, 311.
Peacock, P. R.—(1947) *Brit. med. Bull.*, **4**, 364.
Pullman, A. and Pullman, B.—(1955) ' Cancérisation par les Substances Chimiques et Structure Moléculaire '. Paris (Masson et Cie).
Purves, H. D. and Griesbach, W. E.—(1947) *Brit. J. exp. Path.*, **28**, 46.
Putman, F. L.—(1945) *Dermat. Syph.*, **51**, 221.
Rehn, L.—(1895) *Arch. klin. Chir.*, **50**, 588.
Robertson, J. W. and Clement, K. W.—(1948) *Ohio St. med. J.*, **44**, 280.
Roe, F. J. C.—(1955) *Nature*, **175**, 636.
*Idem* and Salaman, M. H.—(1954) *Brit. J. Cancer*, **8**, 666.—(1955) *Ibid.*, **9**, 177.
Ross, W. C. J.—(1949) *J. chem. Soc.*, p. 183.—(1953) ' Advances in Cancer Research ', **1**, 397. New York (Academic Press).
Russell, B., Green, B. and Wand, L. G. R.—(1948) *Lancet*, ii, 169.
Salaman, M. H. and Roe, F. J. C.—(1953) *Brit. J. Cancer*, **7**, 472.
Sandin, R. B., Melby, R., Hay, A. S., Jones, R. N., Miller, E. C. and Miller, J. A.—(1952) *J. Amer. chem. Soc.*, **74**, 5073.
Sassi, C. and Paruccini, C.—(1954) *Med. d. Lavoro*, **45**, 93 ; *Chem. Abs.*, **48**, 9585.
Schoental, R., Head, M. A. and Peacock, P. R.—(1954) *Brit. J. Cancer*, **8**, 458.
Scott, T. S.—(1952) *Brit. J. industr. Med.*, **9**, 127.
Seligman, A. M., Rutenberg, A. M. and Friedman, O. M.—(1949) *J. nat. Cancer Inst.*, **9**, 261.
Shear, M. J.—(1938) *J. biol. Chem.*, **123**, *Proc.* cviii.
Shimkin, M. B.—(1954) *Cancer*, **7**, 410.
Sissons, H. A.—(1950) *Acta Un. int. Cancer*, **7**, 171.
Smith, H. H. and Srb, A. M.—(1951) *Science*, **114**, 490.
Snegireff, L. S. and Lombard, O. M.—(1951) *Arch. industr. Hyg.*, **4**, 199.
Suntzeff, V., Babcock, R. S. and Loeb, L.—(1940) *Amer. J. Cancer*, **39**, 56.
Spielman, M. A. and Meyer, R. K.—(1939) *J. Amer. chem. Soc.*, **61**, 893.
Spitz, S., Maguigan, W. H. and Dobriner, K.—(1950) *Cancer*, **3**, 789.
Thompson, R. H. S. and Whittaker, V. P.—(1947) *Biochem. J.*, **41**, 342.
Turner, F. C.—(1941) *J. nat. Cancer Inst.*, **2**, 81.
Van Ordstrand, H. S., Hughes, R., DeNardi, J. M. and Carmody, M. G.—(1945) *J. Amer. med. Ass.*, **129**, 1084.
Walpole, A. L., Roberts, D. C., Rose, F. L., Hendry, J. A. and Homer, R. F.—(1954) *Brit. J. Pharmacol.*, **9**, 306.
Walpole, A. L., Williams, M. H. C. and Roberts, D. C.—(1952) *Brit. J. industr. Med.*, **9**, 255.—(1954) *Ibid.*, **11**, 105.—(1955) *Brit. J. Cancer*, **9**, 170.
Weisburger, E. K.—(1955) *J. Amer. chem. Soc.*, **77**, 1914.
*Idem* and Weisburger, J. H.—(1954) *J. org. Chem.*, **19**, 964.—(1955) *Ibid.*, **20**, 1396.
Wieland, H. and Dane, E.—(1933) *Z. physiol. Chem.*, **219**, 240.
Wiley, F. H.—(1938) *J. biol. Chem.*, **124**, 627.
Wilson, R. H., DeEds, F. and Cox, A. J.—(1941) *Cancer Res.*, **1**, 595.—(1947*a*) *Ibid.*, **7**, 444.—(1947*b*) *Ibid.*, **7**, 453.